# Nutritional Inadequacy: Unraveling the Methodological Challenges for the Application of the Probability Approach or the EAR Cut-Point Method—A Pregnancy Perspective

**DOI:** 10.3390/nu13103473

**Published:** 2021-09-29

**Authors:** Foteini Tsakoumaki, Charikleia Kyrkou, Apostolos P. Athanasiadis, Georgios Menexes, Alexandra-Maria Michaelidou

**Affiliations:** 1 Department of Food Science and Technology, School of Agriculture, Faculty of Agriculture, Forestry and Natural Environment, Aristotle University of Thessaloniki, 541 24 Thessaloniki, Greece; foteinitsak@hotmail.com (F.T.); ckyrkou@hotmail.gr (C.K.); 2 3rd Department of Obstetrics and Gynecology, School of Medicine, Aristotle University of Thessaloniki, 541 24 Thessaloniki, Greece; apostolos3435@gmail.com; 3 Department of Field Crops and Ecology, School of Agriculture, Faculty of Agriculture, Forestry and Natural Environment, Aristotle University of Thessaloniki, 541 24 Thessaloniki, Greece; gmenexes@agro.auth.gr

**Keywords:** nutritional (in)adequacy, usual intake, simulated data, bootstrap, percentile distribution, probability approach, EAR cut-point method, point and interval estimation, uncertainty, pregnancy

## Abstract

The aim of this study was to unravel the methodological challenges when exploring nutritional inadequacy, involving 608 healthy pregnant women. The usual intake of twenty-one nutrients was recorded by employing a validated FFQ. Simulated datasets of usual intake were generated, with randomly imposed uncertainty. The comparison between the usual intake and the EAR was accomplished with the probability approach and the EAR cut-point method. Point estimates were accompanied by bootstrap confidence intervals. Bootstrap intervals applied on the risk of inadequacy for raw and simulated data tended in most cases to overlap. A detailed statistical analysis, aiming to predict the level of inadequacy, as well as the application of the EAR cut-point method, along with bootstrap intervals, could effectively be used to assess nutrient inadequacy. However, the final decision for the method used depends on the distribution of nutrient-intake under evaluation. Irrespective of the applied methodology, moderate to high levels of inadequacy, calculated from FFQ were identified for certain nutrients (e.g., vitamins C, B6, magnesium, vitamin A), while the highest were recorded for folate and iron. Considering that micronutrient-poor, obesogenic diets are becoming more common, the underlying rationale may help towards unraveling the complexity characterizing nutritional inadequacies, especially in vulnerable populations.

## 1. Introduction

The appraisal of nutritional inadequacy is of utmost importance, since adequate nutrition is needed before conception and throughout the lifespan [1,2,3,4]. According to International Federation of Gynecology and Obstetrics (FIGO) there is a cycle of passing “health capital” from one generation to the next [5]. Thus, access to adequate food supply and nutrition is inextricably linked to healthy living, growth, and development [4]. In fact, assessment of nutritional inadequacy across different populations is important for nutritional planning and food policy applications, such as the development of food-based dietary guidelines, food fortification and enrichment programs [6].

On a more immediate level, the key principles for the assessment of nutritional inadequacy can be described as follows: (1) collection of food intake data, (2) translation of food intake into nutrient intake, and (3) comparison of the recorded usual intake against reference values [7]. As far as the latter is concerned, several perspectives and methodologies have been developed, across the world. Thorough reviews of the body of literature and principles on this topic have already been published [7,8,9,10] and the interested reader is referred to these outstanding publications in order to be informed on such issues. Among the reference values reported in the literature, Dietary Reference Intakes (DRIs), developed under the auspices of the Institute of Medicine (IOM), are described by four core values: Estimated Average Requirement (EAR), Recommended Dietary Allowance (RDA), Adequate Intake (AI) and safe Upper Level of intake (UL) (The EAR represents the average daily nutrient level estimated to meet the requirements of 50% of healthy individuals in a particular life stage and gender group [11]. The RDA is defined as the EAR plus two standard deviations of the requirement and represents the average daily intake sufficient to meet the needs of nearly all (97%–98%) healthy individuals in a population. The AI is defined as the recommended intake value based on observed or experimentally determined approximations or estimates of nutrient intake by a group of apparently healthy people that are assumed to be adequate. Finally, the UL describes the highest average daily nutrient intake level likely to pose no risk of adverse health effects to almost all individuals in the general population [11]). Of these, EAR is the appropriate DRI to use for evaluating nutrient intakes at the population level [12]. 

In this context, the comparison between the usual intake and the EAR comprises two methods: the “probability approach” (probability of inadequacy) and the “EAR cut-point method” (prevalence of inadequacy) [6,11,13]. By construction, the probability approach is considered more accurate, given the fact that the distribution of requirements is used to determine the risk that the individual’s intake does not meet the EAR value [11]. On the other hand, the EAR cut-point method is simpler and consists of estimating the proportion of population with usual intakes below the EAR, provided that specific assumptions are satisfied [13,14]. For nutrients without a determined EAR the percentage of individuals with intakes below the AI can be calculated. Nevertheless, in this case, the estimates do not refer to the prevalence of inadequacy, but to the likelihood that the usual intake is below the AI [15]. 

Regardless of the method actually chosen to assess the inadequacy, issues related to the uncertainty arising from the assessment of usual intake must also be tackled [11,16]. According to European Food Safety Authority (EFSA) [17], bootstrapping is one of the main solutions to deal with uncertainty. Bootstrapping is a procedure that resamples a single dataset to create many simulated samples without knowing the true distribution of the random variables examined. It is mainly a computation method for producing robust estimates of standard errors and confidence intervals for statistical populations’ parameters, such as the mean and the standard deviation [18,19]. Thus, the bootstrap confidence intervals may contribute to better estimates of the actual inadequacy [20]. 

Screening to identify people at risk is of utmost importance, particularly regarding specific populations, which are simultaneously hard to study and vulnerable to nutrient inadequacy (e.g., pregnant women) [8]. As fetal nutritional environment is sensitive to maternal dietary habits [21], pregnancy is, indeed, an essential stage of the lifecycle, since maternal nutritional status is a strong determinant of both maternal and offspring health [22,23,24]. In particular, it is well-known that micronutrients are involved in all stages of cell growth and differentiation, including cell signaling and protein translation, and are key factors of many enzymes and cell structures. For example, iron inadequacy is linked to maternal anemia and thus, to the increased likelihood of preterm birth, low birth weight and intrauterine growth restriction [25,26], while deficiencies of B-group vitamins are also strong determinants of pregnancy outcome. Thus, placental abruption, preterm deliveries, and other adverse clinical outcomes including preeclampsia and fetal malformations are associated with insufficient intakes of riboflavin, vitamin B6, folate, or cobalamin [27]. Based on the well documented concept that pregnancy can be realized as a key window of opportunity to link early nutrition with long term health, identifying women still at risk remains a scientific issue of vital importance. Therefore, the purpose of this manuscript is to explore the methodological challenges associated with the application of the probability approach and the EAR cut-point method. Bearing in mind that micronutrient-poor, obesogenic diets are becoming more common, this endeavor and the underlying rationale may help toward unraveling the complexity characterizing the assessment of nutritional inadequacies, especially in vulnerable populations. 

## 2. Materials and Methods

### 2.1. Study Population

#### 2.1.1. Participants

Six-hundred and 73 pregnant women (673) were invited to participate, while visiting the 1st Department of Obstetrics and Gynecology, Papageorgiou General Hospital, Thessaloniki, Greece, during the second trimester of pregnancy. All subjects gave their informed consent for inclusion before they participated in the study. The study was conducted according to the guidelines of the Declaration of Helsinki, and approved by the Bioethics Committee of the Medical School, Aristotle University, Thessaloniki, Greece (A19479—26/2/08). 

#### 2.1.2. Exclusion Criteria

Of the 673 women initially enrolled in the investigation, 48 women were excluded for the following reasons: (i) 7 women could not provide appropriate dietary information, (ii) 29 were diagnosed with medical complications that could affect maternal dietary habits, such as diabetes, hypertension, hypercholesterolemia, and coeliac disease, and (iii) 12 were removed due to the inconsistency of answers, as evaluated by the cross-check and summary questions. Furthermore, at a second level, 17 participants with biological improbable intakes (caloric intake greater than 3500 kcal per day) were excluded. This cut-off point was established taking into consideration the Willett’s arbitrary allowable range for women (500 to 3500 kcal per day) [28], as well as the guidelines of EFSA [29]. Application of all the above criteria resulted in a total of 608 women finally included in the study.

### 2.2. Data Collection 

#### 2.2.1. Collection of Demographic/Anthropometric Characteristics and Lifestyle Factors 

Data were collected prior to the antenatal appointment via personal interview [30]. Women were asked to provide information on demographic and anthropometric characteristics. Pre-gestational body mass index (BMI) classification was based on the standards outlined by the World Health Organization (WHO) BMI criteria [31]. The short version of the International Physical Activity Questionnaire (IPAQ) [32] was used for the evaluation of the physical activity status. 

#### 2.2.2. Collection of Dietary Data 

Maternal dietary usual intake was assessed with a Mediterranean oriented, culture-specific Food Frequency Questionnaire (FFQ) that has been previously validated for the pregnant population [30]. Data collection was accomplished via private interview with a registered dietician or a well-trained interviewer.

The conversion of participants’ responses into dietary data was conducted using a Microsoft Excel database [30]. Furthermore, updated information regarding nutrient content and labeling specifications of commercially available food products were taken into account. Approximately 68% of the women reported using supplements (Supplementary Table S1). However, this information was not considered sufficient to be included in the analyses, since various products were reported and a reliable evaluation of the obtained data turned out to be difficult.

### 2.3. Schematic Visualization of the Sequence of Steps Followed in the Present Study

**Figure 1:**
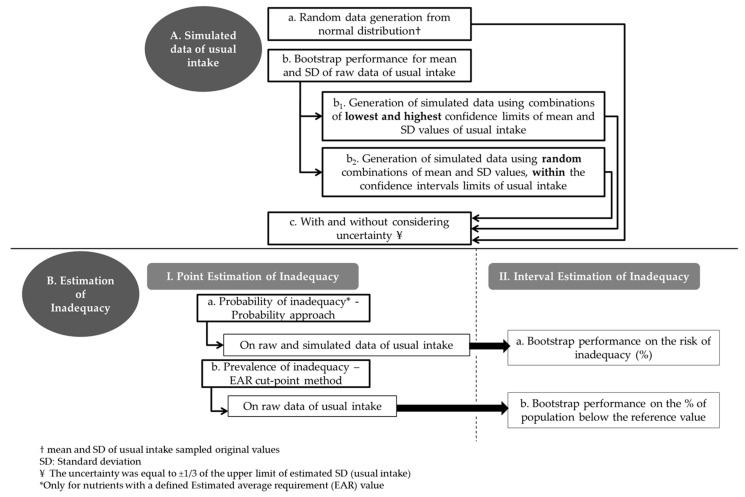
Methodological design for the generation of simulated data of the usual intake (**A**) and the estimation of inadequacy in the studied population (**B**).

The procedure and steps followed in the present study are summarized in Figure 1.

### 2.4. Generation of Simulated Data

Simulated datasets were created according to our methodological design, which is schematically outlined in Figure 1A:

- Step a: Random data (n = 608 cases) from Normal Distribution were generated based on the values of mean and standard deviation (SD) of usual intake sampled original values (**a**).
- Step b: Thirty bias corrected 99% bootstrap confidence intervals (CI) were estimated around the mean and SD of the original data. Each bootstrap run was based on 500 resampling circles (**b**).
- Step b_1_–b_2_: Given the resulted CI from Step b, the lowest (min) and the highest (max) low and upper bounds of the CI, for the mean and the corresponding SD, were selected and used to generate new random normally distributed data sets of usual intake, as in step a. Specifically, one set was based on the combination of the min bound of the mean and the min bound of the corresponding SD. Three other sets were based on the following combinations: min bound of the mean and max bound of the SD, max bound of the mean and min bound of the SD and max bound of mean and max bound of the SD (**b_1_**). In addition, 30 new data sets were generated based on random combinations within the lower-upper bounds of the mean and SD values of usual intake (**b_2_**). A portion of the results is reported in the manuscript.
- Step c. On each of the previously generated data sets an additional degree of uncertainty was “imposed” by randomly adding or subtracting the 1/3 of the upper limit of estimated SD (usual intake), which is an appropriate measure of uncertainty for normally distributed data (**c**).

### 2.5. Assessment of Nutritional Inadequacy 

#### 2.5.1. Measures of Nutrient Inadequacy

Nutrient inadequacy was estimated for protein, carbohydrate and fiber intake, as well as for the following 18 micronutrients: thiamin, riboflavin, niacin, vitamin B6, folate, vitamins B12, C, A, and E, calcium, phosphorus, magnesium, potassium, sodium, zinc, copper, selenium, and iron.

The yardsticks for comparison of the estimations of the assessment of inadequacy, in the present study, focus on the United States (US) and IOM perspectives. The DRIs values proposed by the IOM were used [11,13]. 

#### 2.5.2. Methodologies for the Assessment of Inadequate Intake

The methodological framework for assessing nutrient inadequacy is schematically outlined in Figure 1B.

i. *Probability approach*

The probability of inadequacy of 16 nutrients (Table 1) was evaluated using the probability approach, proposed by Beaton [33]. This approach was applied on log-transformed vitamin E values, since the distribution of raw data was skewed (Supplementary Table S2) [11,33]. The probability approach was not applied on iron based on the fact that too few data were available to simulate an iron requirement distribution for pregnant women and to calculate a SD of the requirements [34,35]. 

Risk curves (Supplementary Figures S1–S4) were constructed in order to associate intake levels to risk levels under the assumed requirement distribution [13,36]. 

To compute the probability of inadequacy on raw and simulated data of usual intake for each nutrient, the NORM. DIST function of MS-Excel was applied, using the EAR and the SD of the requirement, as parameters [13]. The inadequacy of the population, as a point estimation (Figure 1(BIa)), was obtained from the average of individual probabilities and expressed as percentage [11,15]. To provide an interval estimation (Figure 1(BIIa)), bootstrap CIs of the probability of inadequacy estimates were calculated [18]. Each bootstrap run was based on 500 resampling circles at 95% confidence level.

ii. *EAR cut-point method*

The prevalence of inadequacy of 20 nutrients (Table 1) was evaluated using the EAR cut-point method. As such, this method was applied on 16 nutrients (including iron) with a determined EAR value that met the assumption of normality. Iron was included, since in the absence of bleeding or pregnancy only a small quantity of iron is lost [13,34]. For fiber, calcium, potassium and sodium the percentage of individuals with intakes below the AI was calculated [35].

Usual intake values were taken as whole numbers or rounded to the appropriate decimals, depending on the EAR/AI value. The prevalence of inadequacy as a point estimation, i.e., the proportion of individuals below the reference value, was calculated (Figure 1(BIb)). To provide an interval estimation (Figure 1(BIIb)), bootstrap CIs of the final estimate were calculated. Each bootstrap run was based on 500 resampling circles at 95% confidence level.

### 2.6. Statistical Analysis

#### 2.6.1. Descriptive Statistics

For demographic/anthropometric characteristics of pregnant women, continuous variables are presented as mean and SD values, while categorical variables as absolute and relative frequencies (Table 2). For nutrient intakes, mean (SD), median, minimum and maximum values are provided. Furthermore, a detailed percentile distribution of 20 nutrients under study that met the assumption of normality is given in Table 3. As such, vitamin E was excluded.

#### 2.6.2. Generation of Simulated Data

Bias corrected and accelerated (BCa) bootstrap CIs [18] were calculated around mean and SD of usual intake (99% CI based on 500 resampling circles, Figure 1(Ab)). The combination of the derived ranges of mean and SD was used to generate simulated datasets. An additional degree of uncertainty was “imposed” by randomly adding or subtracting the 1/3 of the upper limit of the estimated SD of usual intake.

#### 2.6.3. Interval Estimation of Inadequacy

Intervals estimations of nutritional inadequacy were derived by the application of BCa bootstrap method on the: a. mean probability of inadequacy (95% CI based on 500 resampling circles, Figure 1(BIIa)), and b. percentage of population with intakes below the EAR/AI (95% CI based on 500 resampling circles, Figure 1(BIIb)).

#### 2.6.4. Statistical Tests/Functions and Software Version Used

Normal distribution was checked using skewness and kurtosis (Supplementary Table S2). In the cases where the assumption of normality was not satisfied, values were log-transformed. 

The NORM.DIST function of MS-Excel was applied to compute the probability of inadequacy. The mean probability of inadequacy was expressed as percentage (%). 

All statistical analyses were performed with IBM SPSS v.27.0 (SPSS Inc., Chicago, IL, USA).

## 3. Results and Discussion

### 3.1. Descriptive Features of the Participants

The general characteristics of the 608 participants are given in Table 2. Mean maternal age was 36.5 years. The 65.6% had normal BMI prior to becoming pregnant and 45.9% were high school graduates. Almost 95% had low to moderate physical activity level, while as expected, 85% were non-smokers. General dietary characteristics are given in Supplementary Table S1. Regarding protein intake, expressed as g/kg/day, the mean value (SD) was 1.39 (±0.30) g/kg/day, while mean carbohydrate intake was 226.76 (±38.13) g/day. The contribution of macronutrients to total energy intake is also given in Supplementary Table S1.

### 3.2. Detailed Descriptive Characteristics of Usual Intake as Potential Predictors of the Level of Inadequacy

In this section, the percentile distribution of usual intake, of the population under study, is discussed (Table 3), since this type of descriptive statistics, constitutes a prerequisite for addressing issues relevant to the interpretation of the level of inadequacy [11]. The most interesting aspects of Table 3 are the following: a. the percentile approximately equal to the EAR value may be used to obtain a rough estimation of the percentage of “inadequate” population, and b. the range of percentiles from the EAR to the RDA values may contributes to the identification of individuals not included in the “inadequate” population, but still “at risk” (Supplementary Table S3). 

**Figure 2:**
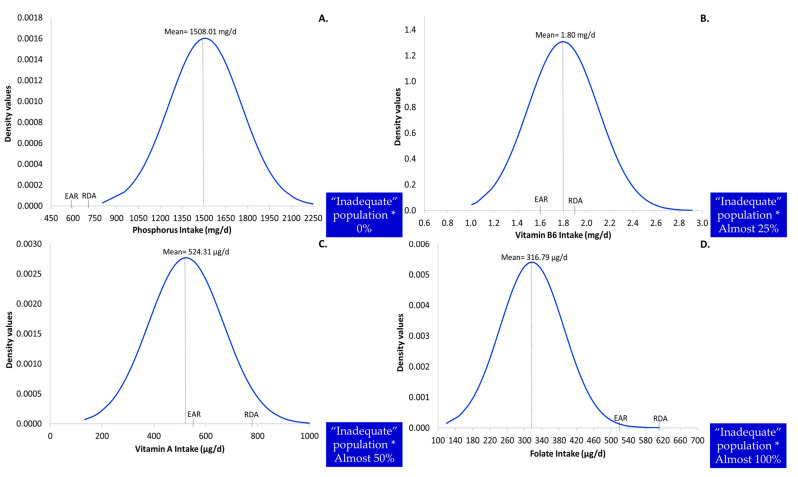
Distribution curve of usual intake for phosphorus (**A**), vitamin B6 (**B**), vitamin A (**C**) and folate (**D**) (*n* = 608). EAR: Estimated Average Requirement; RDA: Recommended Dietary Allowance; * Rough estimation.

Additionally, the illustrated relationship between the usual intake and reference values, i.e., EAR and RDA, is provided in Figure 2, as an alternative screening tool for exploring inadequacy. Thus, from the visual inspection of the indicative diagrams (Figure 2) it can be surmised that the location of the usual intake distribution curve to the right (phosphorus, Figure 2A) or to the left (folate, Figure 2D) of the reference values is translated to 0% or to 100% population risk, respectively, (Figure 2A,B). The greater the shift of the usual intake distribution curve from the right to the left of the reference values, the higher the anticipated level of inadequacy (Figure 2B–D). The 50% of inadequacy is expected when the mean usual intake equals the EAR [11]. 

### 3.3. Documentation for the Generation of Simulated Datasets of Usual Intake 

**Figure 3:**
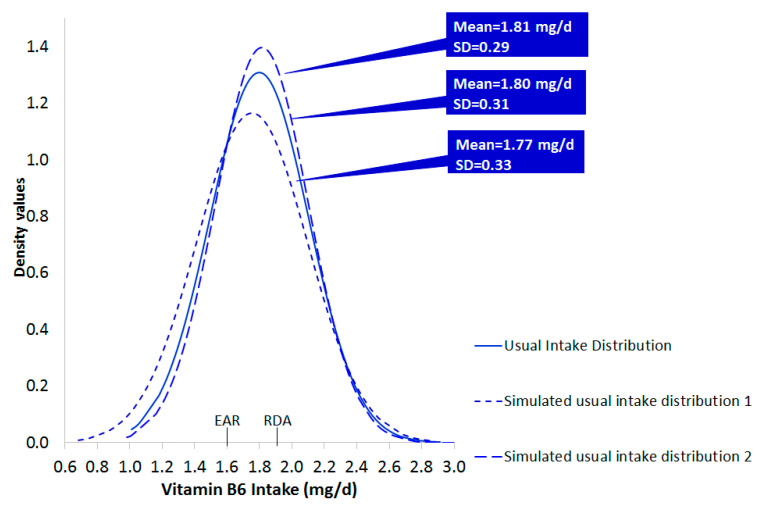
Usual intake distribution of raw and randomly selected simulated data of vitamin B6.

The estimation of usual intake is a pivotal stage in assessing inadequacies [15,37,38,39]. Given the fact that one cannot draw a firm conclusion from one survey [20], the generation of alternative “versions” of usual intake is suggested. The first step toward obtaining a more realistic estimate of the usual intake distribution was the computation of bootstrap CI for the mean and SD of the original observations. Hence, simulated datasets of 608 observations were derived using different combinations of mean and SD, with and without imposing uncertainty. From Table 4, it is apparent that the produced values lay fairly close to the measures of central tendency and dispersion of the raw data. However, as depicted in an excerpt of the derived distributions of usual intake (Figure 3) the predicted values create slightly different distributions, compared to that of raw data, regarding location and shape. Thus, the exploration of nutritional inadequacy, in both conditions (raw and simulated), would be useful, as delineated in the following paragraphs.

### 3.4. Profile of Nutritional Inadequacy

In the paragraphs to follow the profile of nutritional inadequacy will be realized through the application of the probability approach (on raw and simulated data) and the EAR cut-point method (on raw data).

The probability approach, according to the IOM [11], combines two distinct distributions: the requirement distribution, which provides the risk of inadequacy attached to each intake level, and the usual intake distribution, which provides the intake levels and the frequency of each intake in the study population (Supplementary Figures S1–S4). Hence, the mean probability of inadequacy corresponds to the average risk, derived by calculating the risk of inadequacy for each individual in the population under study [7].

When the probability approach was applied on the raw data of usual intake, point estimates, reflecting the mean probability of inadequacy, indicated a zero, for phosphorus, to a fairly low level of inadequacy (<8.7%) for carbohydrate, vitamin B12, copper, selenium, protein, riboflavin and thiamin (Table 5). Higher risk (14.0–30.0%) was estimated for zinc, vitamin C, niacin and vitamin B6, while for magnesium, vitamin A and vitamin E the risk recorded was between 48.9–61.8%. The highest level of inadequacy, under the conditions of this study, corresponds to folate (98%). 

When the probability approach was applied to the model predictions of usual intake, with or without considering the “noise” from sampling variability, a great number of alternate versions of point estimates of risk were produced (Table 5). 

**Figure 4:**
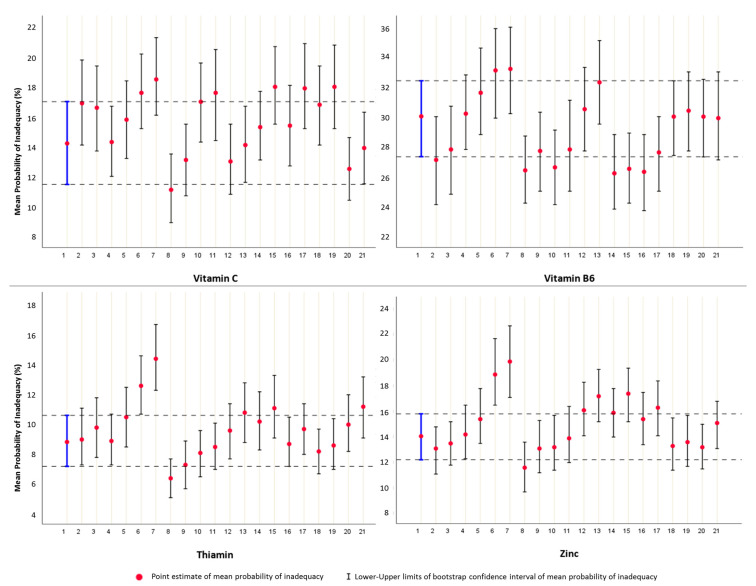
Point estimates and bootstrap confidence intervals for the mean probability of inadequacy (%) for vitamins C, B6, thiamin and zinc. The estimates were calculated for: **1.** usual intake (blue line), and simulated datasets generated by: **2–3.** the mean and standard deviation of the original values, **4–10.** the combinations of lower and upper confidence limits of mean and standard deviation values of usual intakes, **11–21.** random combinations of mean and standard deviation values of usual intakes, within the confidence interval limits of usual intake. Odd numbers: Scenarios without considering the uncertainty into the model; Even numbers: Scenarios with considering the uncertainty into the model.

The first impression from Table 5 is that the values of the generated scenarios tend to overestimate the risk of inadequacy, compared to point estimates derived by the raw data of usual intake. We therefore sought to determine the bootstrap BCa intervals of the final estimates, in raw (Table 5) and simulated data (Supplementary Figures S5–S7), to provide a safer generalization of the level of inadequacy. Interestingly, as we depict in Supplementary Figures S5–S7, the 95% BCa confidence intervals of raw and generated scenarios overlap in most cases, implying that the differences did not reach statistical significance. An excerpt of the derived diagrams is shown in Figure 4. Of note, is that the simulated datasets that did not seem to follow this “overlap pattern rule” were produced using the combinations associated with the greatest degree of uncertainty, i.e., LL of mean-UL of SD of usual intake (e.g., thiamin and zinc in Figure 4).

Keeping in mind that the raw data of usual intake provide only a “snapshot” of the parent population, an interim conclusion that may be drawn from the above comparisons is that the inclusion of bootstrap confidence intervals may contribute to better estimates of the actual inadequacy and should be given to accompany point estimates. 

The comparison between the usual intake and the EAR value may also be accomplished by the EAR cut-point method. Table 6 provides the estimates considering the level of inadequacy for nutrients with a determined EAR or the level of insufficiency for nutrients with an established AI. A substantial proportion of individuals participating in the study met the EAR/AI for phosphorus, carbohydrate, vitamin B12, copper, sodium, selenium, protein, riboflavin, thiamin, niacin, and zinc (<10% of population with intakes below the EAR/AI). The “inadequate” population ranged from 13.70 to 23.52% for vitamins C and B6 and from 50.00 to 59.54% for magnesium and vitamin A. Of the women, 29.93% and 50.16% had insufficient intake of potassium and calcium, while the respective percentage in the case of fiber was 78.95%. Almost all women did not meet the EAR for iron and folate (99.18–100.00%) (Table 6). Consistent with our interim conclusion, the application of bootstrap technique, on the final estimate, was employed. According to Efron [40] “current bootstrap intervals, even nonparametric ones, are usually more accurate than their standard counterparts”.

Irrespective of the method used, low levels expressed either as % below the EAR/AI or as mean probability of inadequacy, were recorded for phosphorus, carbohydrate, vitamin B12, copper, selenium, sodium (AI), protein, riboflavin, thiamin, niacin, and zinc. Moderate levels of inadequacy were estimated for vitamins C and B6, higher for magnesium, vitamin A and E and the highest for folate and iron. The majority of the population had sufficient intake of potassium, while the insufficiency of intake for calcium and fiber was recorded at higher levels.

### 3.5. Comparative Analysis Based on the Construction Framework of the Two Approaches

**Figure 5:**
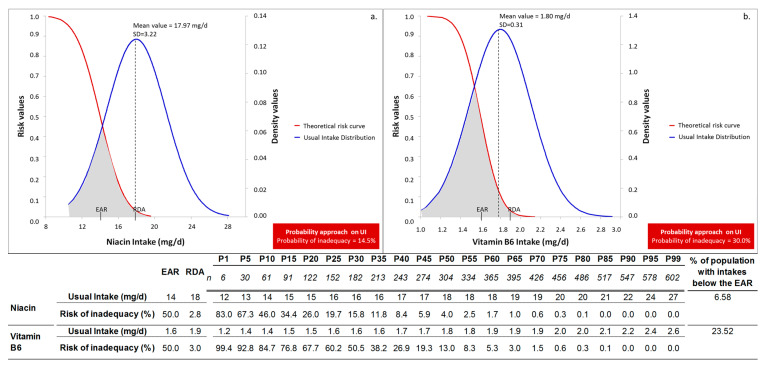
Risk curve and usual intake distributions of the study population for niacin (**a**), and vitamin B6 (**b**), cases of with mean usual intake above the EAR value, but equal/below the RDA value. Risk of inadequacy expresses the corresponding risk attached to each intake; SD: Standard Deviation; UI: Usual Intake; EAR: Estimated Average Requirement; RDA: Recommended Dietary Allowance.

As was already explained, the distribution of usual intake contributes to the determination of the level of inadequacy in both methods, while the distribution of requirement only in the probability approach [14]. This fundamental premise, in conjunction with the previous detailed descriptive characteristics of usual intake, provided in Section 3.2, could explain the differences regarding the construction framework of the two discrete methodological approaches. The combined evaluation of our findings on the profile of inadequacy is presented in Supplementary Table S4. We have decided to render this comparative analysis more conceivable by presenting two selected examples—concerning nutrients with mean usual intake above the EAR value, but equal/below the RDA value (Figure 5), namely the case of niacin and vitamin B6. 

In particular, in the case of niacin, the intake of the individuals between P5 and P10 was equal to EAR value, and therefore, the percentage (%) of population with intakes below the EAR was 6.58%. However, nearly 50% of individuals (P10–P60) were characterized by intakes between 14 and 18 mg/day (Figure 5a), with risk that falls within the range of 46.0% and 1.7%, respectively. Thus, individuals with intakes between the EAR and the RDA, who are still at risk, indeed, contribute to the level of inadequacy recorded by the probability approach, reaching 14.5%. This observation confirms the statement made above (Section 3.2) that the detailed descriptive statistics may be used as a rough estimator for the identification of individuals still “at risk”. 

Noteworthy also, regarding the distribution of usual intake of niacin, was the high risk of inadequacy associated with intakes below 13 mg/day (P5). Indeed, this finding, was more pronounced in another “spread-out” distribution that of vitamin B6. The “inadequate” population for vitamin B6 was 23.52%, since the EAR value was approximately met at P25. However, individuals contributing to this estimate with intakes below/equal to 1.4 mg/day (P10) may present risk > 84.7%, while the intake of 1.2 mg/day is attached to 99.4% risk (Figure 5b). This observation may be critical especially during pregnancy considering that the EAR value of 1.6 mg/day is not the desirable, but the lowest continuing nutrient level that will maintain plasma pyridoxal 5′-phosphate (PLP) levels, at least, at 20 nmol/L [13,41]. Thus, the most striking points emerging from this comparative analysis, refer to the identification of individuals: i. at high risk below the EAR (intakes approximately 75% of EAR), and ii. at moderate risk with intakes between EAR and RDA values (Supplementary Table S4). The greater the mean of usual intake compared to EAR and RDA values, the better the approximation of the level of inadequacy by the EAR cut-point method, and the lesser the individuals identified at high risk level (below 75% EAR). In cases of “spread-out” distributions, especially when intakes are located between the EAR and RDA values, the probability approach may have distinct advantages over the conventional EAR cut-point method. 

### 3.6. Commentary on Our Conceptual Design and Findings: A Contextual Point of View 

The increasing intake of energy-dense but nutrient-poor foods may contribute to food insecurity [5]. Therefore, although a balanced diet can be accessible, micronutrient inadequacies are common, even in developed countries, rendering the appraisal of actual inadequacy a challenging task [3,5,24]. This task may be confronted as a highly complex endeavor, since it comprises many key steps that are prone to uncertainties and discrepancies across the world [6,7,42]. Hence, in order to draw a reliable picture of nutritional status, especially for vulnerable populations, e.g., pregnant women, and to derive optimal solutions, the harmonization of different perspectives and methodologies is of high priority [6,7,42,43].

**Figure 6:**
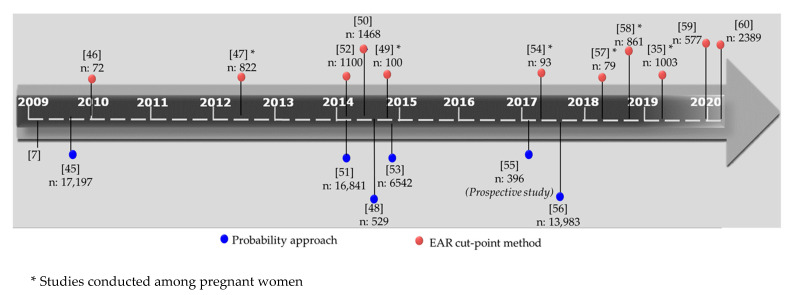
Timeline of selected studies that have applied the probability approach and the EAR cut-point method, since 2009.

In the present study, as already mentioned in Section 2.5.1, the yardsticks proposed by the IOM were used as reference values. In this context, a comprehensive review of the literature on nutritional inadequacy, focusing on US and IOM perspectives, indicated that the EAR cut-point method gains ground over the probability approach. Indeed, as presented in Figure 6, there is a shift toward adopting this straightforward procedure, since 2009, as it possesses alluring features, due to its appealing simplicity and ease of implementation [7,35,44,45,46,47,48,49,50,51,52,53,54,55,56,57,58,59,60].

Consistent with this tendency, studies conducted on pregnant women assessed the inadequacy using the EAR cut-point method (Figure 6). Hence, the critical assessment of our results against these published data is, to a degree, allowable since all studies comprised in Table 7 concern pregnant women, and use the IOM yardsticks. Tabacchi et al. [7] highlight the importance of the variation that can occur when different yardsticks are implemented to assess nutritional inadequacies. To provide a more legitimate comparison, data shown in Table 7 correspond to dietary intakes from food items only. The release of the final estimates using BCas confidence intervals may facilitate the comparison, among studies; BCas confidence intervals, dealing with uncertainty may alleviate the heterogeneity associated with other methodological aspects, e.g., the dietary intake assessment tools and the food composition databases used to translate food to nutrient intake. In this frame, based on the produced bootstrap BCas intervals, our findings were found comparable to the results of the selected studies (Table 7), at least as far as the following nutrients are concerned: thiamin, niacin, riboflavin, vitamins B6, B12 and C, iron, copper and zinc. It is noteworthy that similar levels of iron inadequacy have been reported for the Greek population by Hatzopoulou et al. [49]. Additionally, similar levels of folate inadequacy were reported in a study conducted in Spain [47].

The comparison among studies would have been further facilitated if the release of the final estimates as bootstrap confidence intervals had been adopted as a common strategy for the evaluation of the actual inadequacy.

### 3.7. Strengths and Limitations

The key point in determining inadequacies centers on the assessment of usual intake [15]. With this aim, we used a Mediterranean oriented semi-quantitative FFQ, developed and validated by our research group [30]. Food frequency questionnaires could be considered to be suitable tools to assess inadequacies of micronutrients [61] and are frequently used, particularly for sample sizes greater than 500 participants (Figure 6).

To achieve greater accuracy, quality, and adequacy of data collection [45,62], the dietary recording was accomplished via private interview with a registered dietician or a well-trained interviewer. The fact that the “precise frequency” version [30] was adopted adds further credence to the information gathered for the 21 nutrients assessed, in the current study.

However, supplements were not considered to be part of the analytical framework, an issue that could be faced as a limitation. According to Mensink [42], the impact of supplements is dependent on the supplement formulation, the frequency of use and the levels of micronutrient intakes of those taking supplements. Our research effort has focused on the examination of the nutritional inadequacies of dietary intakes from food items only, since the data collected regarding: the range of supplements taken, frequency of consumption, duration of intake, and doses involved were not considered sufficient, in order to be included in the analyses.

An interesting methodological feature of the present study was the application of data simulation on usual intake, with or without the combination of random noise (uncertainty), which allowed the experimentation with different assumptions or decisions. As was reported in the literature [63,64], the stochastic mechanism of data generation gives usually more clear insights, especially, into complex systems and provides some kind of proof that the system or the model is better understood. Thus, even though models are “idealizations” or simplified versions of reality and therefore cannot possibly replace it in every case, they are used to simulate reality.

The outcome of the present study is further strengthened by the following facts: a. the applied methodological framework is based on the guidelines elaborated by IOM [11,13] and recognized as the preferable methodology within the EURopean micronutrient RECommendations Aligned network (EURRECA) [6], b. the implementation of the probability approach on a sample population greater than 100 individuals, since according to the literature the mean probability of inadequacy may not accurately mirror the true prevalence of inadequate intakes for groups of less than about 100 individuals [61,65], and c. the additional methodological procedures proposed in this study, such as the construction of confidence intervals on the final estimates, which was based on the bias corrected and accelerated method. This method according to Efron [40] provides intervals that are usually more accurate than their standard counterparts. However, although bootstrap is a versatile technique which allows the estimation of the sampling distribution of almost any statistical index, the bootstrap results may depend on the representativeness of the examined sample. Consequently, in cases where the representativeness of the sample in hand is not guaranteed and supported, the findings based on bootstrap analysis should be interpreted with caution [40]. In the present study, the sample population was representative with respect to the mean age of childbearing and the education level of women 30–39 years old, in Greece [66,67].

This study was approached from a pregnancy perspective. The fact that dietary intake was assessed only during the 2nd trimester, may be considered to be a limitation, since, the portrait of dietary intake may not be typical for the entire pregnancy period; nevertheless, previous studies have suggested that there is stability in dietary intakes across trimesters [57,68]. As stated in the Introduction Section, according to FIGO there is a cycle of passing “health capital” from one generation to the next [5]. Thus, the assessment of inadequacies during pregnancy is inextricably linked to healthy living, growth and development [4].

## 4. Conclusions

**Figure 7:**
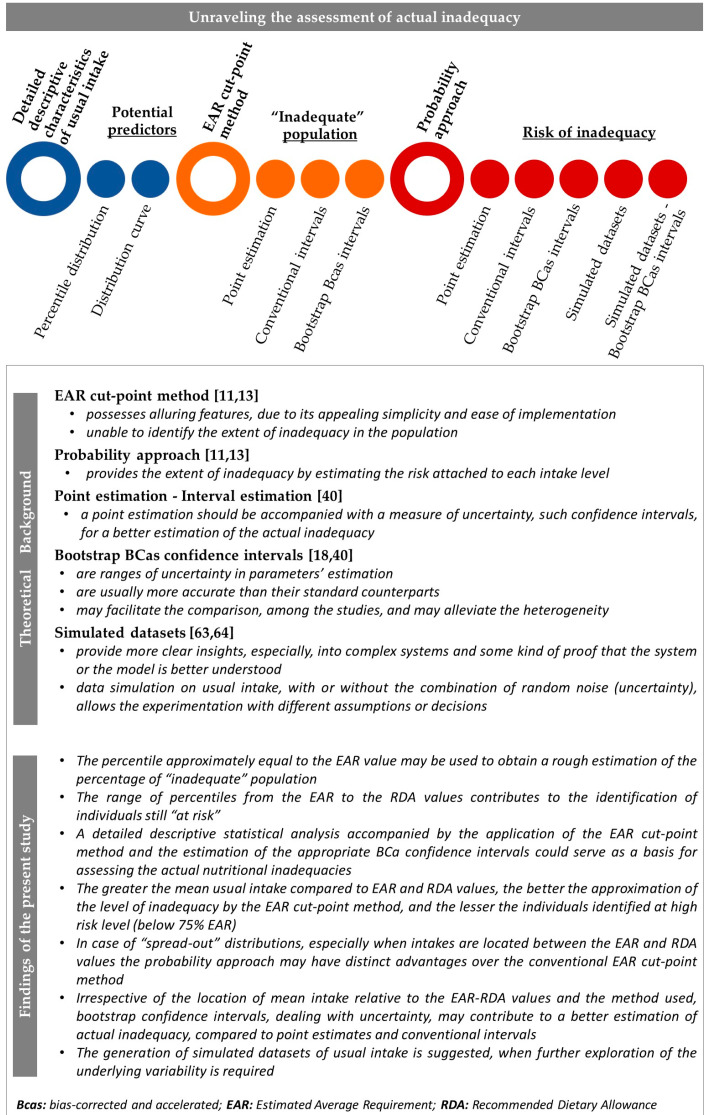
Key conclusions of the present study.

The key points of the main methodological findings of the present study, based on the adopted experimental framework of data collection and analyses, are illustrated in Figure 7.

The estimated risk of inadequacy corresponding to each nutrient intake level, unravels “hidden” high risk groups of varying extent of actual inadequacy. This observation is of considerable importance, especially in cases where nutritional inadequacies are not pronounced, i.e., at low and moderate levels of inadequacy, as is depicted in Figure 6 and Supplementary Table S4. This postulation of “hidden inadequacy” is of clinical relevance, since reducing pregnant women with suboptimal micronutrient intakes still “at risk”, may reduce the likelihood of adverse fetal programming and future disease.

Targeting optimal pregnancy with the minimum probable adverse consequences for the offspring, presents, among others, a challenging task to attain adequate micronutrient intake through diet. In view that nutrient requirements are best met through balanced dietary patterns, which incorporate nutrient-dense foods, issues related to diet during pregnancy remain interesting and deserve further study. With this aim, a comprehensive mapping of dietary patterns has been developed and becomes particularly helpful in promoting public health messages and introducing food policy guidelines, throughout this crucial stage of the lifecycle.

## Figures and Tables

**Table 1:** Applied methods for estimating the nutrient inadequacy for the macro- and micronutrients under study.

		Probability Approach	EAR Cut-Point Method	Comments
1	**Protein**	+	+	
2	**Carbohydrate**	+	+	
3	**Fiber**		+	AI
4	**Thiamin**	+	+	
5	**Riboflavin**	+	+	
6	**Niacin**	+	+	
7	**Vitamin B6**	+	+	
8	**Folate**	+	+	
9	**Vitamin B12**	+	+	
10	**Vitamin C**	+	+	
11	**Vitamin A**	+	+	
12	**Vitamin E**	+		Skewed distribution
13	**Calcium**		+	AI
14	**Phosphorus**	+	+	
15	**Magnesium**	+	+	
16	**Potassium**		+	AI
17	**Sodium**		+	AI
18	**Zinc**	+	+	
19	**Copper**	+	+	
20	**Selenium**	+	+	
21	**Iron**		+	Not established SD

EAR: Estimated Average Requirement; AI: Adequate intake; SD: standard deviation.

**Table 2:** General characteristics of the 608 pregnant women.

Demographic/Anthropometric Characteristics	Mean (SD)
Maternal age (year)	36.50 (3.77)
	***n* (%)**
**Pre-pregnancy BMI**	
Underweight (BMI < 18.5 kg/m^2^)	26 (4.3)
Normal (BMI 18.5–24.9 kg/m^2^)	399 (65.6)
Overweight (BMI 25–29.9 kg/m^2^)	123 (20.2)
Obese (BMI > 30.0 kg/m^2^)	60 (9.9)
**Education**	
Tertiary education (universities)	130 (21.4)
Tertiary technical education	98 (16.1)
Post secondary non-tertiary education	76 (12.5)
High school	279 (45.9)
Lower secondary education school	25 (4.1)
Physical activity level *	
Low activity	473 (77.8)
Moderate activity	101 (16.6)
High activity	34 (5.6)
**Smoking during pregnancy**	
Occasional or daily smokers	91 (15.0)
Non-smokers	517 (85.0)

* As derived by the IPAQ [32], SD: standard deviation, BMI: body mass index.

**Table 3:** Reference Values and detailed percentile distribution of usual intake (*n* = 608).

	EAR/AI			P1	P5	P10	P15	P20	P25	P30	P35	P40	P45	P50	P55	P60	P65	P70	P75	P80	P85	P90	P95	P99	“Inadequate” population *
			*n*	6	30	61	91	122	152	182	213	243	274	304	334	365	395	426	456	486	517	547	578	602	
		RDA																							
**Phosphorus (mg/day)**	580	700		982	1121	1194	1248	1292	1333	1369	1402	1426	1467	1496	1526	1553	1583	1630	1669	1719	1777	1845	1947	2145	<1%
**Carbohydrate (g/day)**	135	175		145	170	182	188	195	199	204	209	214	219	225	229	233	238	244	249	256	268	280	300	321	<1%
**Vitamin B12 (μg/day)**	2.2	2.6		2.2	2.9	3.3	3.7	3.8	4.0	4.2	4.4	4.6	4.7	4.8	5.0	5.2	5.3	5.4	5.6	5.8	6.2	6.5	6.8	8.1	<1%
**Copper (μg/day)**	800	1000		794	934	1007	1052	1097	1137	1172	1217	1254	1289	1327	1375	1419	1475	1528	1584	1642	1706	1781	1931	2196	1–5%
**Selenium (μg/day)**	49	60		44	51	56	59	61	63	66	67	69	70	72	73	75	77	79	81	83	87	90	98	114	1–5%
**Protein (g/kg/day)**	0.88	1.1		0.75	0.93	1.02	1.07	1.14	1.18	1.24	1.27	1.31	1.36	1.39	1.42	1.46	1.52	1.55	1.60	1.66	1.70	1.76	1.89	2.11	1–5%
**Riboflavin (mg/day)**	1.2	1.4		1.0	1.2	1.4	1.5	1.6	1.7	1.7	1.8	1.9	1.9	2.0	2.1	2.2	2.2	2.3	2.4	2.5	2.6	2.7	2.9	3.2	1–5%
**Thiamin (mg/day)**	1.2	1.4		1.0	1.2	1.2	1.3	1.4	1.4	1.5	1.5	1.6	1.6	1.6	1.7	1.7	1.8	1.8	1.9	2.0	2.0	2.1	2.2	2.5	1–5%
**Niacin (mg/day)**	14	18		12	13	14	15	15	16	16	16	17	17	18	18	18	19	19	20	20	21	22	24	27	5–10%
**Zinc (mg/day)**	9.5	11		8.0	9.0	9.5	9.9	10.1	10.4	10.7	10.9	11.1	11.2	11.4	11.7	11.9	12.2	12.4	12.7	13.0	13.5	13.9	14.7	15.6	5–10%
**Vitamin C (mg/day)**	70	85		32	51	60	72	81	88	94	102	109	116	126	137	144	152	164	174	182	194	218	246	306	10–15%
**Vitamin B6 (mg/day)**	1.6	1.9		1.2	1.4	1.4	1.5	1.5	1.6	1.6	1.6	1.7	1.7	1.8	1.8	1.9	1.9	1.9	2.0	2.0	2.1	2.2	2.4	2.6	20–25%
**Magnesium (mg/day)**	300	360		197	227	243	252	260	266	271	278	287	293	299	307	315	323	331	344	354	365	381	405	456	50–55%
**Vitamin A (μg/day)**	550	770		240	307	343	381	403	423	444	465	481	492	507	531	552	571	591	615	637	667	720	776	922	55–60%
**Folate (μg/day)**	520	600		169	207	229	241	255	265	277	285	292	303	311	322	333	341	350	358	370	388	413	448	519	>99%
**Iron (mg/day)**	22			7	8	9	9	10	10	10	10	10	11	11	11	12	12	12	13	13	14	14	16	17	>99%
**Sodium (g/day)**	1.5 **			1.4	1.6	1.7	1.8	1.9	2.0	2.0	2.1	2.1	2.2	2.2	2.3	2.3	2.4	2.4	2.5	2.6	2.6	2.8	3.0	3.4	1–5%
**Potassium (g/day)**	2.9 **			2.1	2.4	2.5	2.6	2.7	2.8	2.9	2.9	3.0	3.0	3.1	3.2	3.2	3.3	3.4	3.5	3.6	3.7	3.8	4.1	4.7	25–30%
**Calcium (mg/day)**	1000 **			448	597	693	753	803	839	874	913	935	970	998	1026	1060	1101	1141	1175	1225	1270	1351	1484	1662	50–55%
**Fiber (g/day)**	28 **			12	15	17	18	18	19	20	21	21	22	22	23	24	25	26	26	28	29	30	33	39	75–80%

P: Percentile, EAR: Estimated Average Requirement. The intakes equal to the EAR correspond to 50% risk, RDA: Recommended Dietary Allowance. The intakes equal to the RDA correspond to negligible risk, * Rough estimation—the percentile approximately equal to the EAR value may be used to obtain a rough estimation of the percentage of “inadequate” population, ** AI: Adequate Intake, Vitamin E was excluded since it did not meet the assumption of normality.

**Table 4:** Mean and standard deviation of usual intake of raw data used to derive bootstrap confidence intervals.

			(99%) Bootstrap CI			
			Mean Value		SD Value	
	Mean	SD	LL	UL	LL	UL
**Protein (g/kg/day)**	1.39	0.30	1.37	1.42	0.28	0.30
**Carbohydrate (g/day)**	226.76	38.13	223.57	230.72	36.14	39.87
**Thiamin (mg/day)**	1.67	0.33	1.64	1.69	0.32	0.35
**Riboflavin (mg/day)**	2.02	0.50	1.98	2.04	0.47	0.53
**Niacin (mg/day)**	17.97	3.22	17.67	18.26	3.08	3.41
**Vitamin B6 (mg/day)**	1.80	0.31	1.77	1.82	0.28	0.33
**Folate (μg/day)**	316.79	73.72	309.55	322.93	69.01	79.64
**Vitamin B12 (μg/day)**	4.88	1.24	4.80	4.98	1.14	1.33
**Vitamin C (mg/day)**	134.05	61.11	129.94	138.64	58.15	68.64
**Vitamin A (μg/day)**	524.31	143.87	512.24	535.25	134.37	152.92
Vitamin E (mg/day) *	1.05	0.10	1.04	1.06	0.09	0.10
**Phosphorus (mg/day)**	1508.01	248.73	1493.07	1523.85	235.50	260.52
**Magnesium (mg/day)**	306.35	55.07	300.97	310.78	52.07	58.55
**Zinc (mg/day)**	11.60	1.71	11.45	11.75	1.60	1.80
**Copper (μg/day)**	1374.14	312.48	1349.93	1397.06	290.72	330.37
**Selenium (μg/day)**	72.80	13.91	71.65	74.15	13.15	14.64

* log transformed. 500 bootstrap replicates were applied at 99% confidence interval level. LL: Lower Limit. UL: Upper Limit. CI: Confidence Interval. SD: Standard deviation.

**Table 5:** Mean Probability of Inadequacy on raw and simulated data of usual intake.

	Mean Probability of Inadequacy (%)																				
	On UI (LL–UL of BCa CI)		On Simulated Data																		
				Combinations of Lowest and Highest CI Limits of Mean/SD Values of UI								Random Combinations within the CI Limits of Mean/SD Values of UI									
		Mean/SD of UI		LL of Mean/LL SD of UI		LL of Mean/UL SD of UI		UL of Mean/LL SD of UI		UL of Mean/UL SD of UI		1 *		2 *		3 *		4 *		5 *	
		**a**	**b**	**a**	**b**	**a**	**b**	**a**	**b**	**a**	**b**	**a**	**b**	**a**	**b**	**a**	**b**	**a**	**b**	**a**	**b**
**Phosphorus**	0.0 (0–0)	0.0	0.0	0.0	0.0	0.0	0.0	0.0	0.0	0.0	0.0	0.0	0.0	0.0	0.0	0.0	0.0	0.0	0.0	0.0	0.0
**Carbohydrate**	1.1 (0.7–1.5)	1.4	2.0	1.7	2.3	2.1	2.5	1.0	1.7	1.9	2.3	1.6	2.0	1.7	1.9	2.4	2.5	1.7	1.9	1.7	2.4
**Vitamin B12**	1.2 (0.7–1.8)	2.7	3.6	1.4	2.4	2.7	3.3	0.9	1.1	1.5	2.0	1.5	2.1	1.9	1.8	0.8	1.2	2.6	3.4	2.3	2.9
**Copper**	2.2 (1.6–2.8)	5.6	6.3	3.2	3.6	5.4	6.8	2.9	3.6	4.4	4.6	2.8	3.4	3.8	4.5	4.0	4.6	4.4	5.6	6.7	7.7
**Selenium**	4.3 (3.2–5.4)	5.1	6.3	4.8	5.6	8.6	9.7	3.4	4.1	5.3	6.0	5.7	6.7	4.1	4.9	4.4	4.9	4.9	5.9	5.6	6.9
**Protein**	4.5 (3.4–5.6)	4.0	5.3	4.2	4.9	5.2	5.8	3.9	4.7	4.7	5.6	4.6	5.2	4.3	5.7	4.1	5.6	4.9	5.7	4.1	5.0
**Rivoflavin**	5.0 (3.7–6.5)	5.1	6.9	5.1	5.3	9.3	9.4	3.9	5.7	6.1	7.5	4.3	5.7	7.2	8.4	5.5	6.1	5.0	5.9	4.5	6.0
**Thiamin**	8.7 (7.1–10.5)	8.9	9.7	8.8	10.4	12.5	14.3	6.3	7.2	8.0	8.4	9.9	11.1	8.6	9.6	8.1	8.5	9.5	10.7	10.1	11.0
**Zinc**	14.0 (12.1–15.7)	13.0	13.4	14.1	15.3	18.8	19.8	11.5	13.0	13.1	13.8	15.3	16.2	13.1	15.0	15.8	17.3	13.2	13.5	16.0	17.1
**Vitamin C**	14.2 (11.5–17)	16.9	16.6	14.3	15.8	17.6	18.5	11.1	13.1	17.0	17.6	13.0	14.1	12.5	13.9	15.3	18.0	16.8	18.0	15.4	17.9
**Niacin**	14.5 (12.8–16)	16.2	17.2	16.4	18.0	20.6	21.7	11.4	13.2	12.6	14.1	16.0	17.4	13.9	14.7	16.2	16.5	16.7	17.2	13.5	14.0
**Vitamin B6**	30.0 (27.3–32.4)	27.1	27.8	30.2	31.6	33.1	33.2	26.4	27.7	26.6	27.8	30.5	32.3	30.0	30.4	26.3	27.6	26.2	26.5	30.0	29.9
**Magnesium**	48.9 (45.8–51.8)	43.7	44.8	47.9	47.2	46.4	47.2	42.2	42.1	43.4	43.6	44.3	44.6	51.1	51.2	42.8	43.7	45.0	45.9	50.6	50.2
**Vitamin A**	57.2 (54.8–59.7)	56.4	56.3	57.9	57.2	59.0	58.7	54.5	54.5	53.7	53.9	55.6	54.6	53.8	58.7	52.7	53.4	54.4	54.1	55.0	54.7
		a	b	a	b	a	b	a	b	a	b	a	b	a	b	a	b	a	b	a	b
**Vitamin E ^¥^**	61.8 (59.4–64.4)	59.8	59.0	63.8	63.4	62.0	61.7	59.7	60.2	54.9	54.4	55.3	55.2	63.1	62.4	59.5	58.7	61.4	61.2	58.4	58.0
**Folate**	98.0 (97.2–98.8)	99.2	99.0	99.1	99.0	98.3	97.9	98.4	98.0	98.2	97.6	99.4	99.1	98.8	98.3	99.2	98.9	98.6	98.3	99.0	98.8

* 1–5: random combinations of mean-SD within the bootstrap intervals, ¥ log tranformed, UI: usual intake, LL: Lower Limit, UL: Upper Limit, BCa CI: Bias corrected and accelerated bootstrap confidence intervals of the probability of inadequacy (500 bootstrap replicates were applied at 95% CI level), CI: Confidence Intervals, SD: Standard deviation, a: without considering the uncertainty into the model, b: considering uncertainty equal to ±1/3 of the upper limit of estimated SD (usual intake) into the model.

**Table 6:** Prevalence of inadequacy expressed as point estimates and BCa confidence intervals on the usual intake of study population (*n* = 608).

	EAR/AI	% of Population with Intakes below the EAR/AI (LL-UL of BCa CI)
**Phosphorus (mg/day)**	580	0.00 (JPY)
**Carbohydrate (g/day)**	135	0.16 (0.0–0.7)
**Vitamin B12 (μg/day)**	2.2	0.82 (0.3–1.7)
**Copper (μg/day)**	800	0.99 (0.3–2.0)
**Selenium (μg/day)**	49	2.96 (1.2–4.7)
**Protein (g/kg/day)**	0.88	3.13 (1.6–5.5)
**Riboflavin (mg/day)**	1.2	3.13 (1.7–5.6)
**Thiamin (mg/day)**	1.2	4.28 (2.8–6.2)
**Niacin (mg/day)**	14	6.58 (4.8–9.8)
**Zinc (mg/day)**	9.5	9.54 (6.6–15.0)
**Vitamin C (mg/day)**	70	13.70 (10.9–19.4)
**Vitamin B6 (mg/day)**	1.6	23.52 (19.8–28.9)
**Magnesium (mg/day)**	300	50.00 (45.3–62.9)
**Vitamin A (μg/day)**	550	59.54 (55.4–69.6)
**Folate (μg/day)**	520	99.18 (98.2–99.9)
**Iron (mg/day)**	22	100.00 (JPY)
**Sodium (g/day)**	1.5 *	1.32 (0.5–2.4)
**Potassium (g/day)**	2.9 *	29.93 (23.3–36.7)
**Calcium (mg/day)**	1000 *	50.16 (47.1–61.2)
**Fiber (g/day)**	28 *	78.95 (76.6–81.9)

EAR: Estimated Average Requirement; * AI: Adequate Intake; LL: Lower Limit; UL: Upper Limit; BCa CI: Bias corrected and accelerated bootstrap confidence intervals of the prevalence of inadequacy (500 bootstrap replicates were applied at 95%); CI: Confidence Interval; JPY Not applicable.

**Table 7:** Data regarding the percentage of pregnant population with intakes below the EAR/AI as reported by selected studies.

				% of Population with Intakes below the EAR/AI													
Reference	Country	Record of Usual Intake	Extraction of Nutrient Values	Thiamin	Niacin	Riboflavin	Vitamin B6	Vitamin B12	Folate	Vitamin C	Vitamin A	Fe	Mg	Ca	P	Cu	Zn
[47] ◊	Spain	FFQ	USDA †						99.6	14.4	4.6	67.9					0.0
[49] ◊	Greece	3DRs	Food processor Software						87.2			97.9	83.0	55.3			
[54]	USA	3DRs	Nutrition Data System for Research software						66			89		28			
[57] * ◊	Canada	3DRs	NFCT	7.6		1.3	32.9	3.8	60.8	22.8	17.7	89.9	19.0	13.9		1.3	8.9
[58]	Canada	FRs *(3d)*	Food processor Software †	4.2	0.0	0.4	24.5	1.1	66.8	7.2	9.8	95.2	15.8	10.5	0.0		16.9
[35] ◊	USA	2DRs	USDA †¤	11.5	2.8	5.0	25.4	2.4	35.8	24.7	27.7	83.8	53.3	21.2		5.4	21.5
**Present study** **(LL–UL of BCa CI)**	**Greece**	**FFQ**	**	**4.28** **(2.8–6.2)**	**6.58** **(4.8–9.8)**	**3.13** **(1.7–5.6)**	**23.52** **(19.8–28.9)**	**0.82** **(0.3–2.0)**	**99.18** **(98.2–99.9)**	**13.70** **(10.9–19.4)**	**59.54** **(55.4–69.6)**	**100.00** **(¥)**	**50.00** **(45.3–62.9)**	**50.16** **(47.1–61.2)**	**0.00** **(¥)**	**0.99** **(0.3–2.0)**	**9.54** **(6.6–15.0)**

EAR: Estimated Average Requirement; AI: Adequate Intake; USA: United States of America; FFQ: Food Frequency Questionnaire; DRs: 24hrs Dietary Recalls; FR: Food Records; 3d: 3 days; USDA: United States Department of Agriculture, NFCT: National Food Composition Tables, LL: Lower Limit; UL: Upper Limit; BCa CI: Bias corrected and accelerated bootstrap confidence intervals of the prevalence of inadequacy; CI: Confidence Interval, * data refer to the 2nd trimester, ◊ The role of dietary supplements in meeting nutritional requirements was also evaluated, † Data from National Food Composition Tables (NFCT) were also incorporated, ¤ Nutrient content and labeling specifications of commercially available food products were also taken into account, ** The nutrient composition of food items was derived from: (a) the “Food Composition Tables and Composition of Greek Cooked Food and Dishes”; (b) the food composition database developed at the Department of Social Medicine of the University of Crete and (c) the USDA food composition Database for Standard Reference, ¥ Not applicable.

## Supplementary Materials

The following are available online at https://www.mdpi.com/article/10.3390/nu13103473/s1, Figure S1: Risk curve and usual intake distributions of the study population for phosphorus, carbohydrate, vitamin B12, and copper, Figure S2: Risk curve and usual intake distributions of the study population for selenium, protein, riboflavin, and thiamin, Figure S3: Risk curve and usual intake distributions of the study population for zinc, vitamin C, niacin, and vitamin B6, Figure S4: Risk curve and usual intake distributions of the study population for magnesium, vitamin A, and folate, Figure S5: Point estimates and bootstrap confidence intervals for the mean probability of inadequacy (%) for carbohydrates, vitamin B12, copper, selenium, protein and riboflavin. The estimates were calculated for: 1. usual intake (blue line), and simulated datasets generated by: 2–3. the mean and standard deviation of the original values, 4–10. the combinations of lower and upper confidence limits of mean and standard deviation values of usual intakes, 11–21. random combinations of mean and standard deviation values of usual intakes, within the confidence interval limits of usual intake. Odd numbers: Scenarios without considering the uncertainty into the model; Even numbers: Scenarios with considering the uncertainty into the model; Figure S6: Point estimates and bootstrap confidence intervals for the mean probability of inadequacy (%) for thiamin, zinc, vitamin C, niacin, vitamin B6 and magnesium. The estimates were calculated for: 1. usual intake (blue line), and simulated datasets generated by: 2–3. the mean and standard deviation of the original values, 4–10. the combinations of lower and upper confidence limits of mean and standard deviation values of usual intakes, 11–21. random combinations of mean and standard deviation values of usual intakes, within the confidence interval limits of usual intake. Odd numbers: Scenarios without considering the uncertainty into the model; Even numbers: Scenarios with considering the uncertainty into the model. Figure S7: Point estimates and bootstrap confidence intervals for the mean probability of inadequacy (%) for vitamin A, vitamin E and folate. The estimates were calculated for: 1. usual intake (blue line), and simulated datasets generated by: 2–3. the mean and standard deviation of the original values, 4–10. the combinations of lower and upper confidence limits of mean and standard deviation values of usual intakes, 11–21. random combinations of mean and standard deviation values of usual intakes, within the confidence interval limits of usual intake. Odd numbers: Scenarios without considering the uncertainty into the model; Even numbers: Scenarios with considering the uncertainty into the model. Table S1: General dietary characteristics of the 608 pregnant women, Table S2: Descriptive statistics of usual intake for the 21 nutrients explored in the present study (*n* = 608). Table S3. Detailed percentile distribution of usual intake (*n* = 608). Table S4. Corresponding risk attached to each intake in the percentile distribution.
