# Supplementary material for: Nutritional Inadequacy: Unraveling the Methodological Challenges for the Application of the Probability Approach or the EAR Cut-Point Method—A Pregnancy Perspective
Source: Nutrients. 2021 Sep 29;13(10):3473. doi: 10.3390/nu13103473 (PMC8538604; doi:10.3390/nu13103473)
Supplement: Supplementary file 1 [file nutrients-13-03473-s001.zip › nutrients-1305891-Supplementary_Tables.pdf]

Supplementary table 1. General dietary characteristics of the 608 pregnant women

Dietary characteristics	Mean (SD)
Energy (kcal)	2068.40 (226.90)
Protein (g/kg/d)	1.39 (0.30)
Protein (g/d)	88.40 (10.54)
Total lipid (g/d)	92.10 (11.85)
Carbohydrate (g/d)	226.76 (38.13)
% Energy from total protein	17.16 (1.81)
% Energy from total lipid	40.14 (3.52)
% Energy from carbohydrate	43.71 (4.37)
**Dietary supplement intake**	**n (%)**
Yes	418 (68.8)
No	190 (31.3)

SD: standard deviation

Supplementary table 2. Descriptive statistics of usual intake for the twenty-one nutrients explored in the present study (n=608)

	Min	Max	Mean	Median	SD	CV (%)	Skewness	Kurtosis
**Protein (g/kg/d)**	0.50	2.41	1.39	1.39	0.30	21.16	0.17	0.01
**Carbohydrate (g/d)**	114.20	332.69	226.76	225.42	38.13	16.81	0.32	-0.13
**Fiber (g/d)**	6.36	47.73	23.06	22.46	5.55	24.08	0.59	0.78
**Thiamin (mg/d)**	0.97	3.03	1.67	1.63	0.33	20.00	0.43	0.11
**Riboflavin (mg/d)**	0.86	3.63	2.02	2.00	0.50	24.95	0.16	-0.44
**Niacin (mg/d)**	10.53	28.13	17.97	17.67	3.22	17.94	0.54	0.36
**Vitamin B6 (mg/d)**	1.01	2.91	1.80	1.78	0.31	16.98	0.49	0.16
**Folate (μg/d)**	119.16	610.91	316.79	311.36	73.72	23.27	0.53	0.62
**Vitamin B12 (μg/d)**	1.54	9.74	4.88	4.85	1.24	25.41	0.31	0.66
**Vitamin C (mg/d)**	10.29	377.48	134.05	126.32	61.11	45.59	0.71	0.41
**Vitamin A (μg/d)**	133.23	1030.90	524.31	507.31	143.87	27.44	0.46	0.32
**Vitamin E (mg/d)**	6.51	26.60	11.45	10.87	2.72	23.78	1.34	2.86
**Calcium (mg/d)**	288.22	1891.83	1014.74	997.91	257.95	25.42	0.31	0.15
**Phosphorus (mg/d)**	803.54	2251.81	1508.01	1495.99	248.73	16.49	0.28	-0.14
**Magnesium (mg/d)**	184.46	504.45	306.35	299.32	55.07	17.98	0.54	0.24
**Potassium (g/d)**	1.80	5.00	3.15	3.10	0.52	16.58	0.50	0.29
**Sodium (g/d)**	1.20	4.10	2.25	2.20	0.42	18.63	0.61	1.08
**Zinc (mg/d)**	7.21	16.57	11.60	11.45	1.71	14.72	0.25	-0.22
**Copper (μg/d)**	618.73	2872.47	1374.14	1326.76	312.48	22.74	0.64	0.51
**Selenium (μg/d)**	39.31	125.51	72.80	71.57	13.91	19.10	0.50	0.62
**Iron (mg/d)**	6.12	19.53	11.35	10.94	2.17	19.16	0.76	0.60

SD: Standard deviation; CV: Coefficient of variation

Supplementary Table 3. Detailed percentile distribution of usual intake (n=608).

	EAR/AI	RDA		P1	P5	P10	P15	P20	P25	P30	P35	P40	P45	P50	P55	P60	P65	P70	P75	P80	P85	P90	P95	P99	*"Inadequate" population **
			n	*6*	*30*	*61*	*91*	*122*	*152*	*182*	*213*	*243*	*274*	*304*	*334*	*365*	*395*	*426*	*456*	*486*	*517*	*547*	*578*	*602*	
**Phosphorus (mg/d)**	580	700		982	1121	1194	1248	1292	1333	1369	1402	1426	1467	1496	1526	1553	1583	1630	1669	1719	1777	1845	1947	2145	<1%
**Carbohydrate (g/d)**	135	175		145	170	182	188	195	199	204	209	214	219	225	229	233	238	244	249	256	268	280	300	321	<1%
**Vitamin B12 (µg/d)**	2.2	2.6		2.2	2.9	3.3	3.7	3.8	4.0	4.2	4.4	4.6	4.7	4.8	5.0	5.2	5.3	5.4	5.6	5.8	6.2	6.5	6.8	8.1	<1%
**Copper (µg/d)**	800	1000		794	934	1007	1052	1097	1137	1172	1217	1254	1289	1327	1375	1419	1475	1528	1584	1642	1706	1781	1931	2196	1-5%
**Selenium (µg/d)**	49	60		44	51	56	59	61	63	66	67	69	70	72	73	75	77	79	81	83	87	90	98	114	1-5%
**Protein (g/kg/d)**	0.88	1.1		0.75	0.93	1.02	1.07	1.14	1.18	1.24	1.27	1.31	1.36	1.39	1.42	1.46	1.52	1.55	1.60	1.66	1.70	1.76	1.89	2.11	1-5%
**Riboflavin (mg/d)**	1.2	1.4		1.0	1.2	1.4	1.5	1.6	1.7	1.7	1.8	1.9	1.9	2.0	2.1	2.2	2.2	2.3	2.4	2.5	2.6	2.7	2.9	3.2	1-5%
**Thiamin (mg/d)**	1.2	1.4		1.0	1.2	1.2	1.3	1.4	1.4	1.5	1.5	1.6	1.6	1.6	1.7	1.7	1.8	1.8	1.9	2.0	2.0	2.1	2.2	2.5	1-5%
**Niacin (mg/d)**	14	18		12	13	14	15	15	16	16	16	17	17	18	18	18	19	19	20	20	21	22	24	27	5-10%
**Zinc (mg/d)**	9.5	11		8.0	9.0	9.5	9.9	10.1	10.4	10.7	10.9	11.1	11.2	11.4	11.7	11.9	12.2	12.4	12.7	13.0	13.5	13.9	14.7	15.6	5-10%
**Vitamin C (mg/d)**	70	85		32	51	60	72	81	88	94	102	109	116	126	137	144	152	164	174	182	194	218	246	306	10-15%
**Vitamin B6 (mg/d)**	1.6	1.9		1.2	1.4	1.4	1.5	1.5	1.6	1.6	1.6	1.7	1.7	1.8	1.8	1.9	1.9	1.9	2.0	2.0	2.1	2.2	2.4	2.6	20-25%
**Magnesium (mg/d)**	300	360		197	227	243	252	260	266	271	278	287	293	299	307	315	323	331	344	354	365	381	405	456	50-55%
**Vitamin A (µg/d)**	550	770		240	307	343	381	403	423	444	465	481	492	507	531	552	571	591	615	637	667	720	776	922	55-60%
**Folate (µg/d)**	520	600		169	207	229	241	255	265	277	285	292	303	311	322	333	341	350	358	370	388	413	448	519	>99%
**Iron (mg/d)**	22			7	8	9	9	10	10	10	10	10	11	11	11	12	12	12	13	13	14	14	16	17	>99%
**Sodium (g/d)**	1.5**			1.4	1.6	1.7	1.8	1.9	2.0	2.0	2.1	2.1	2.2	2.2	2.3	2.3	2.4	2.4	2.5	2.6	2.6	2.8	3.0	3.4	1-5%
**Potassium (g/d)**	2.9**			2.1	2.4	2.5	2.6	2.7	2.8	2.9	2.9	3.0	3.0	3.1	3.2	3.2	3.3	3.4	3.5	3.6	3.7	3.8	4.1	4.7	25-30%
**Calcium (mg/d)**	1000**			448	597	693	753	803	839	874	913	935	970	998	1026	1060	1101	1141	1175	1225	1270	1351	1484	1662	50-55%
**Fiber (g/d)**	28**			12	15	17	18	18	19	20	21	21	22	22	23	24	25	26	26	28	29	30	33	39	75-80%

P: Percentile

EAR: Estimated Average Requirement. The intakes equal to the EAR correspond to 50% risk.

RDA: Recommended Dietary Allowance. The intakes equal to the RDA correspond to negligible risk.

*Rough estimation

** AI: Adequate Intake.

Vitamin E was excluded since it did not meet the assumption of normality.

Population still "at risk" (Rough estimation) - The range of percentiles from the EAR to the RDA values may contributes to the identification of individuals not included in the "inadequate" population, but still "at risk".

Supplementary table 4. Corresponding risk attached to each intake in the percentile distribution.

		75% EAR	EAR	RDA		P1	P5	P10	P15	P20	P25	P30	P35	P40	P45	P50	P55	P60	P65	P70	P75	P80	P85	P90	P95	P99	% of population with intakes below the EAR	Mean Probability of Inadequacy (%)
					n	*6*	*30*	*61*	*91*	*122*	*152*	*182*	*213*	*243*	*274*	*304*	*334*	*365*	*395*	*426*	*456*	*486*	*517*	*547*	*578*	*602*		
Phosphorus*	Usual Intake (mg/d)	435	580	700		982	1121	1194	1248	1292	1333	1369	1402	1426	1467	1496	1526	1553	1583	1630	1669	1719	1777	1845	1947	2145	0.0	
	Risk of inadequacy (%)	99.4	50.0	1.9		0.0	0.0	0.0	0.0	0.0	0.0	0.0	0.0	0.0	0.0	0.0	0.0	0.0	0.0	0.0	0.0	0.0	0.0	0.0	0.0	0.0		0.0
Carbohydrate*	Usual Intake (mg/d)	101	135	175		145	170	182	188	195	199	204	209	214	219	225	229	233	238	244	249	256	268	280	300	321	0.2	
	Risk of inadequacy (%)	95.2	50.0	2.4		31.1	4.0	1.1	0.5	0.2	0.1	0.0	0.0	0.0	0.0	0.0	0.0	0.0	0.0	0.0	0.0	0.0	0.0	0.0	0.0	0.0		1.1
Vitamin B12*	Usual Intake (μg/d)	1.7	2.2	2.6		2.2	2.9	3.3	3.7	3.8	4.0	4.2	4.4	4.6	4.7	4.8	5.0	5.2	5.3	5.4	5.6	5.8	6.2	6.5	6.8	8.1	0.8	
	Risk of inadequacy (%)	99.4	50.0	3.5		50.0	0.0	0.0	0.0	0.0	0.0	0.0	0.0	0.0	0.0	0.0	0.0	0.0	0.0	0.0	0.0	0.0	0.0	0.0	0.0	0.0		1.2
Copper*	Usual Intake (μg/d)	600	800	1000		794	934	1007	1052	1097	1137	1172	1217	1254	1289	1327	1375	1419	1475	1528	1584	1642	1706	1781	1931	2196	1.0	
	Risk of inadequacy (%)	95.2	50.0	4.8		52.0	13.2	4.2	1.8	0.7	0.2	0.1	0.0	0.0	0.0	0.0	0.0	0.0	0.0	0.0	0.0	0.0	0.0	0.0	0.0	0.0		2.2
Selenium*	Usual Intake (μg/d)	37	49	60		44	51	56	59	61	63	66	67	69	70	72	73	75	77	79	81	83	87	90	98	114	3.0	
	Risk of inadequacy (%)	99.4	50.0	1.2		84.6	31.2	8.3	1.8	0.5	0.2	0.0	0.0	0.0	0.0	0.0	0.0	0.0	0.0	0.0	0.0	0.0	0.0	0.0	0.0	0.0		4.3
Protein*	Usual Intake (g/kg/d)	0.66	0.88	1.1		0.75	0.93	1.02	1.07	1.14	1.18	1.24	1.27	1.31	1.36	1.39	1.42	1.46	1.52	1.55	1.60	1.66	1.70	1.76	1.89	2.11	3.1	
	Risk of inadequacy (%)	98.1	50.0	1.9		89.1	31.5	9.9	3.6	0.6	0.2	0.0	0.0	0.0	0.0	0.0	0.0	0.0	0.0	0.0	0.0	0.0	0.0	0.0	0.0	0.0		4.5
Rivoflavin*	Usual Intake (mg/d)	0.9	1.2	1.4		1.0	1.2	1.4	1.5	1.6	1.7	1.7	1.8	1.9	1.9	2.0	2.1	2.2	2.2	2.3	2.4	2.5	2.6	2.7	2.9	3.2	3.1	
	Risk of inadequacy (%)	99.4	50.0	4.8		95.2	37.0	8.8	1.5	0.1	0.0	0.0	0.0	0.0	0.0	0.0	0.0	0.0	0.0	0.0	0.0	0.0	0.0	0.0	0.0	0.0		5.0
Thiamin*	Usual Intake (mg/d)	0.9	1.2	1.4		1.0	1.2	1.2	1.3	1.4	1.4	1.5	1.5	1.6	1.6	1.6	1.7	1.7	1.8	1.8	1.9	2.0	2.0	2.1	2.2	2.5	4.3	
	Risk of inadequacy (%)	99.4	50.0	4.8		95.2	63.7	36.2	19.0	6.9	2.7	0.9	0.4	0.1	0.0	0.0	0.0	0.0	0.0	0.0	0.0	0.0	0.0	0.0	0.0	0.0		8.7
Niacin**	Usual Intake (mg/d)	11	14	18		12	13	14	15	15	16	16	16	17	17	18	18	18	19	19	20	20	21	22	24	27	6.6	
	Risk of inadequacy (%)	95.2	50.0	2.8		83.0	67.3	46.0	34.4	26.0	19.7	15.8	11.8	8.4	5.9	4.0	2.5	1.7	1.0	0.6	0.3	0.1	0.0	0.0	0.0	0.0		14.5
Zinc*	Usual Intake (mg/d)	7.1	9.5	11		8.0	9.0	9.5	9.9	10.1	10.4	10.7	10.9	11.1	11.2	11.4	11.7	11.9	12.2	12.4	12.7	13.0	13.5	13.9	14.7	15.6	9.5	
	Risk of inadequacy (%)	99.4	50.0	5.7		94.3	70.7	51.3	35.5	26.5	18.5	10.7	7.5	4.8	3.3	2.0	1.1	0.5	0.2	0.1	0.0	0.0	0.0	0.0	0.0	0.0		14.0
Vitamin C*	Usual Intake (mg/d)	53	70	85		32	51	60	72	81	88	94	102	109	116	126	137	144	152	164	174	182	194	218	246	306	13.7	
	Risk of inadequacy (%)	99.4	50.0	1.6		100.0	99.7	92.2	41.0	6.4	0.5	0.0	0.0	0.0	0.0	0.0	0.0	0.0	0.0	0.0	0.0	0.0	0.0	0.0	0.0	0.0		14.2
Vitamin B6**	Usual Intake (mg/d)	1.2	1.6	1.9		1.2	1.4	1.4	1.5	1.5	1.6	1.6	1.6	1.7	1.7	1.8	1.8	1.9	1.9	1.9	2.0	2.0	2.1	2.2	2.4	2.6	23.5	
	Risk of inadequacy (%)	99.4	50.0	3.0		99.4	92.8	84.7	76.8	67.7	60.2	50.5	38.2	26.9	19.3	13.0	8.3	5.3	3.0	1.5	0.6	0.3	0.1	0.0	0.0	0.0		30.0
Magnesium***	Usual Intake (mg/d)	225	300	360		197	227	243	252	260	266	271	278	287	293	299	307	315	323	331	344	354	365	381	405	456	50.0	
	Risk of inadequacy (%)	99.4	50.0	2.3		100.0	99.3	97.1	94.4	91.1	87.3	83.2	76.9	66.9	58.7	50.9	41.3	31.3	22.5	14.8	7.1	3.5	1.5	0.3	0.0	0.0		48.9
Vitamin A***	Usual Intake (μg/d)	413	550	770		240	307	343	381	403	423	444	465	481	492	507	531	552	571	591	615	637	667	720	776	922	59.5	
	Risk of inadequacy (%)	89.4	50.0	2.3		99.8	98.6	97.0	93.8	91.0	87.6	83.2	78.0	73.5	70.2	65.1	56.8	49.4	42.6	35.4	27.6	21.6	14.3	6.1	2.0	0.0		57.2
Folate***	Usual Intake (μg/d)	390	520	600		169	207	229	241	255	265	277	285	292	303	311	322	333	341	350	358	370	388	413	448	519	99.2	
	Risk of inadequacy (%)	99.4	50.0	6.2		100.0	100.0	100.0	100.0	100.0	100.0	100.0	100.0	100.0	100.0	100.0	100.0	100.0	100.0	99.9	99.9	99.8	99.4	98.0	91.7	50.7		98.0

Population still "at risk"

Mean Intake Value

P: Percentile
EAR: Estimated Average Requirement. The intakes equal to the EAR correspond to 50% risk.
RDA: Recommended Dietary Allowance. The intakes equal to the RDA correspond to negligible risk.
Vitamin E was excluded since it did not meet the assumption of normality.

*: Mean Intake Value > EAR, RDA
**: EAR < Mean Intake Value ≤ RDA
***: Mean Intake Value < EAR, RDA
