# Supplementary material for: Nutritional Inadequacy: Unraveling the Methodological Challenges for the Application of the Probability Approach or the EAR Cut-Point Method—A Pregnancy Perspective
Source: Nutrients. 2021 Sep 29;13(10):3473. doi: 10.3390/nu13103473 (PMC8538604; doi:10.3390/nu13103473)
Supplement: Supplementary file 1 [file nutrients-13-03473-s001.zip › Tsakoumaki et al. Supplementary_Figures.pdf]

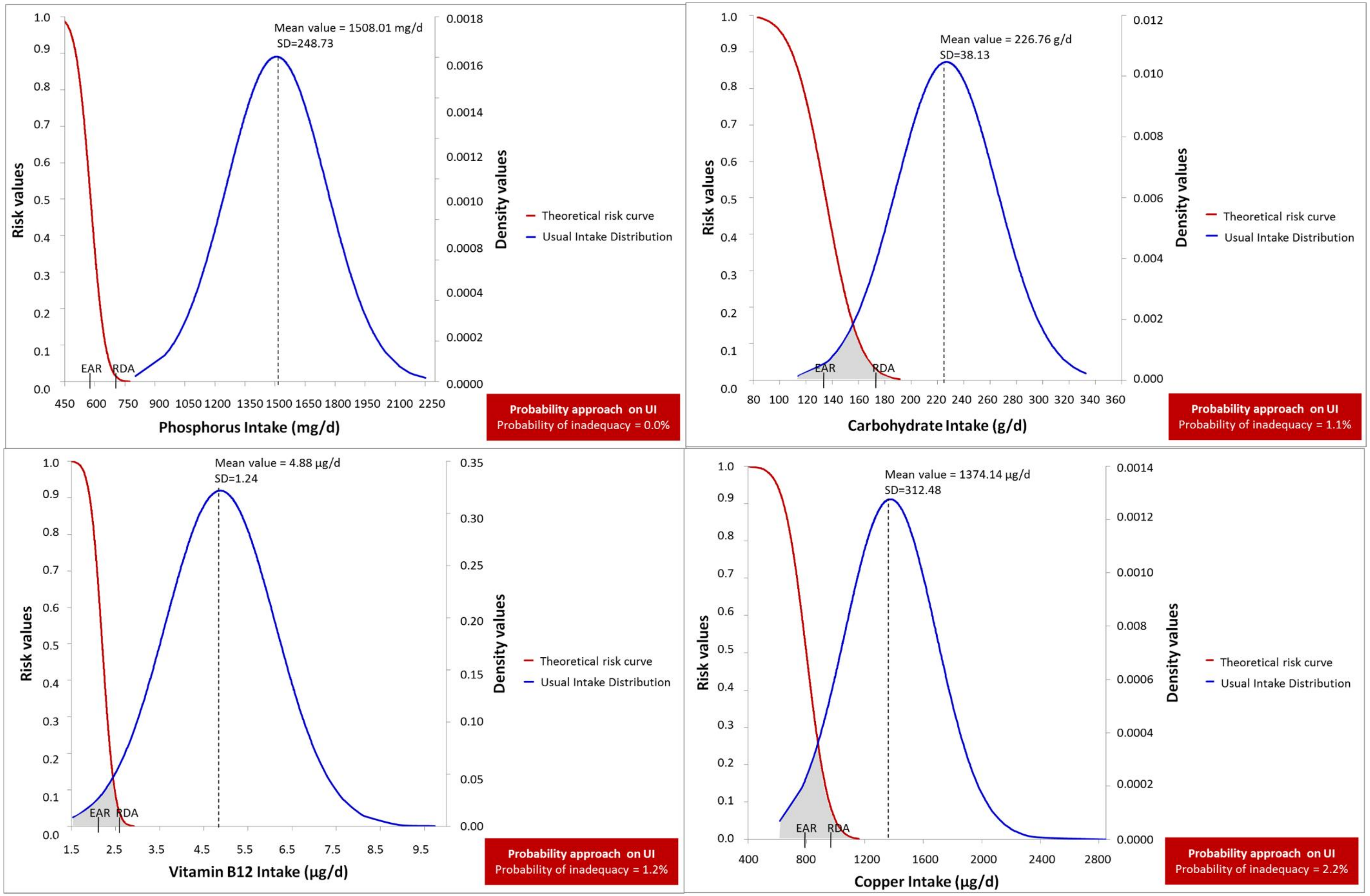

EAR: Estimated Average Requirement; RDA: Recommended Dietary Allowance; UI: Usual Intake

Supplementary figure 1. Risk curve and usual intake distributions of the study population for phosphorus, carbohydrate, vitamin B12, and copper (Charts are sorted in ascending order according to the estimated probability of inadequacy, n=608).

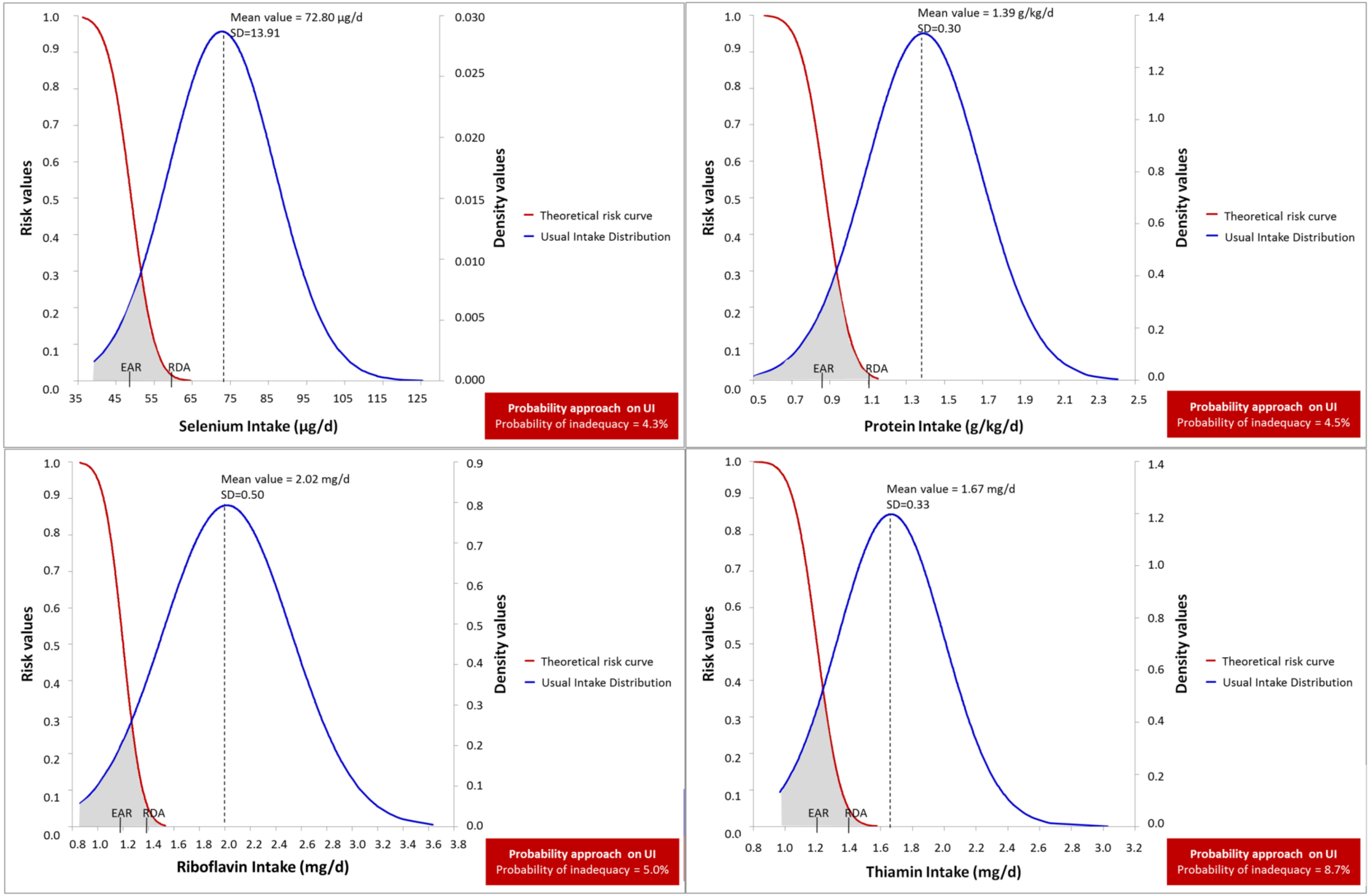

EAR: Estimated Average Requirement; RDA: Recommended Dietary Allowance; UI: Usual Intake

Supplementary figure 2. Risk curve and usual intake distributions of the study population for selenium, protein, riboflavin, and thiamin (Charts are sorted in ascending order according to the estimated probability of inadequacy, n=608).

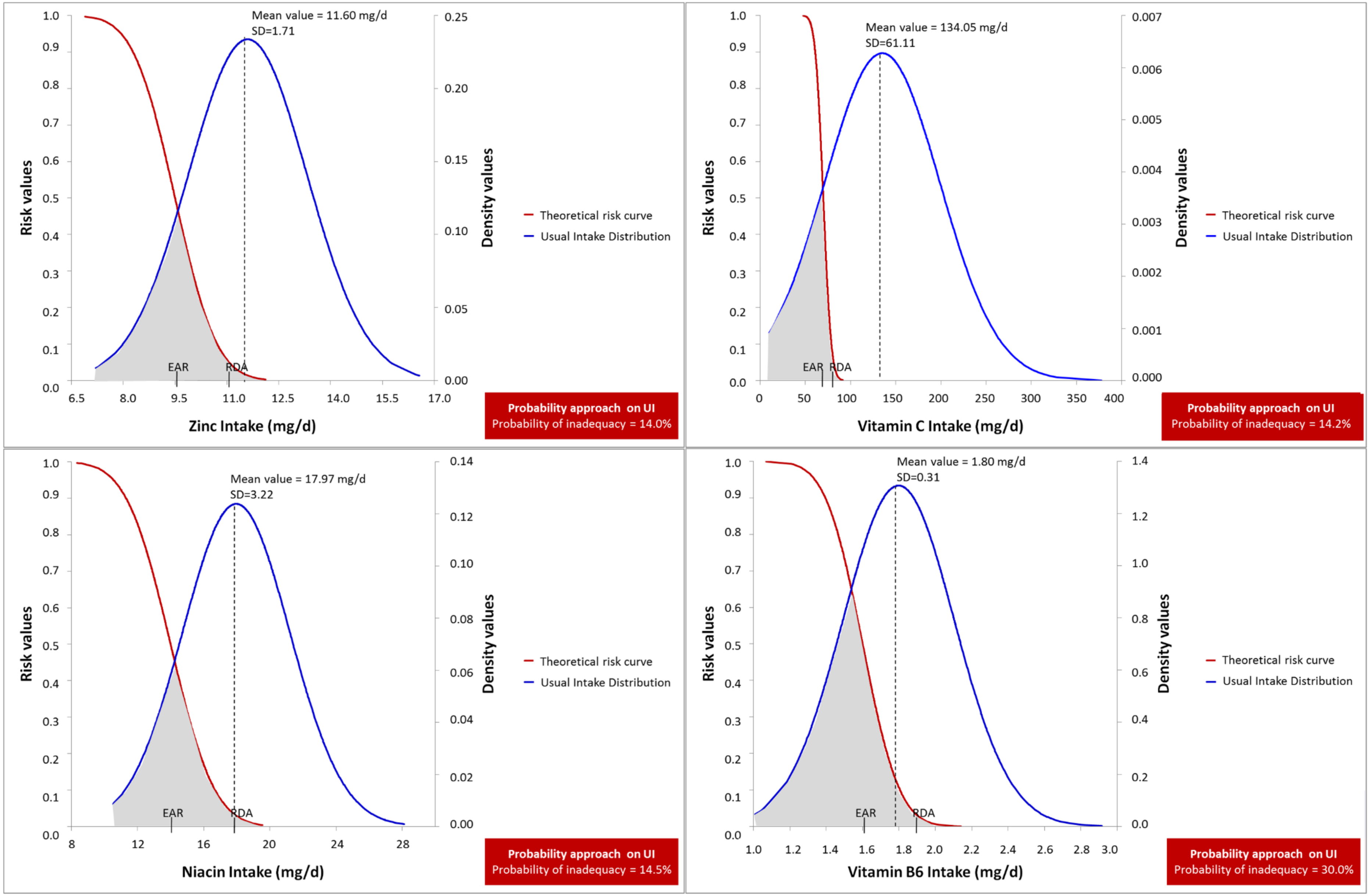

EAR: Estimated Average Requirement; RDA: Recommended Dietary Allowance; UI: Usual Intake

Supplementary figure 3. Risk curve and usual intake distributions of the study population for zinc, vitamin C, niacin, and vitamin B6 (Charts are sorted in ascending order according to the estimated probability of inadequacy, n=608).

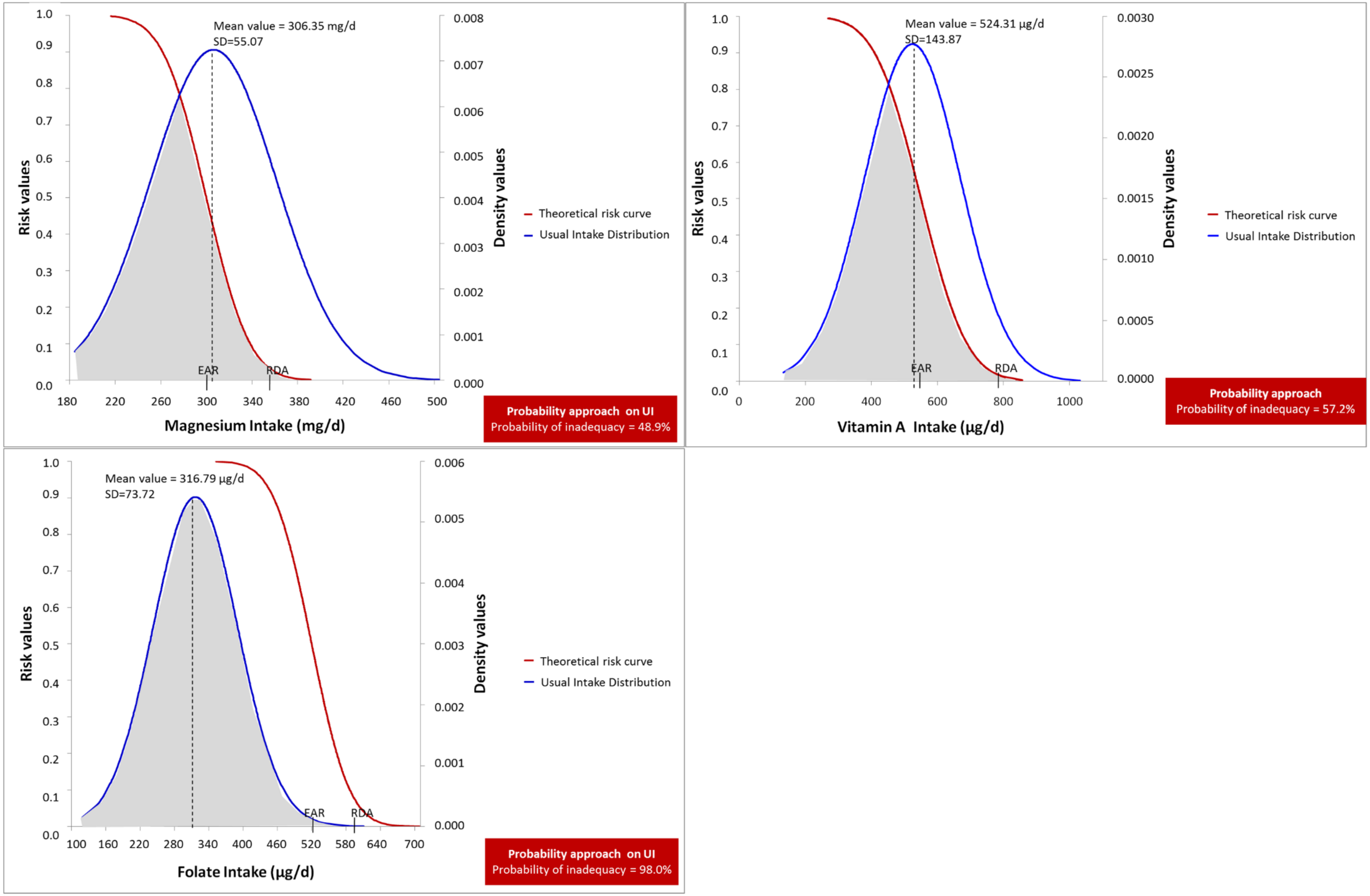

EAR: Estimated Average Requirement; RDA: Recommended Dietary Allowance; UI: Usual Intake

Supplementary figure 4. Risk curve and usual intake distributions of the study population for magnesium, vitamin A, and folate (Charts are sorted in ascending order according to the estimated probability of inadequacy, n=608).

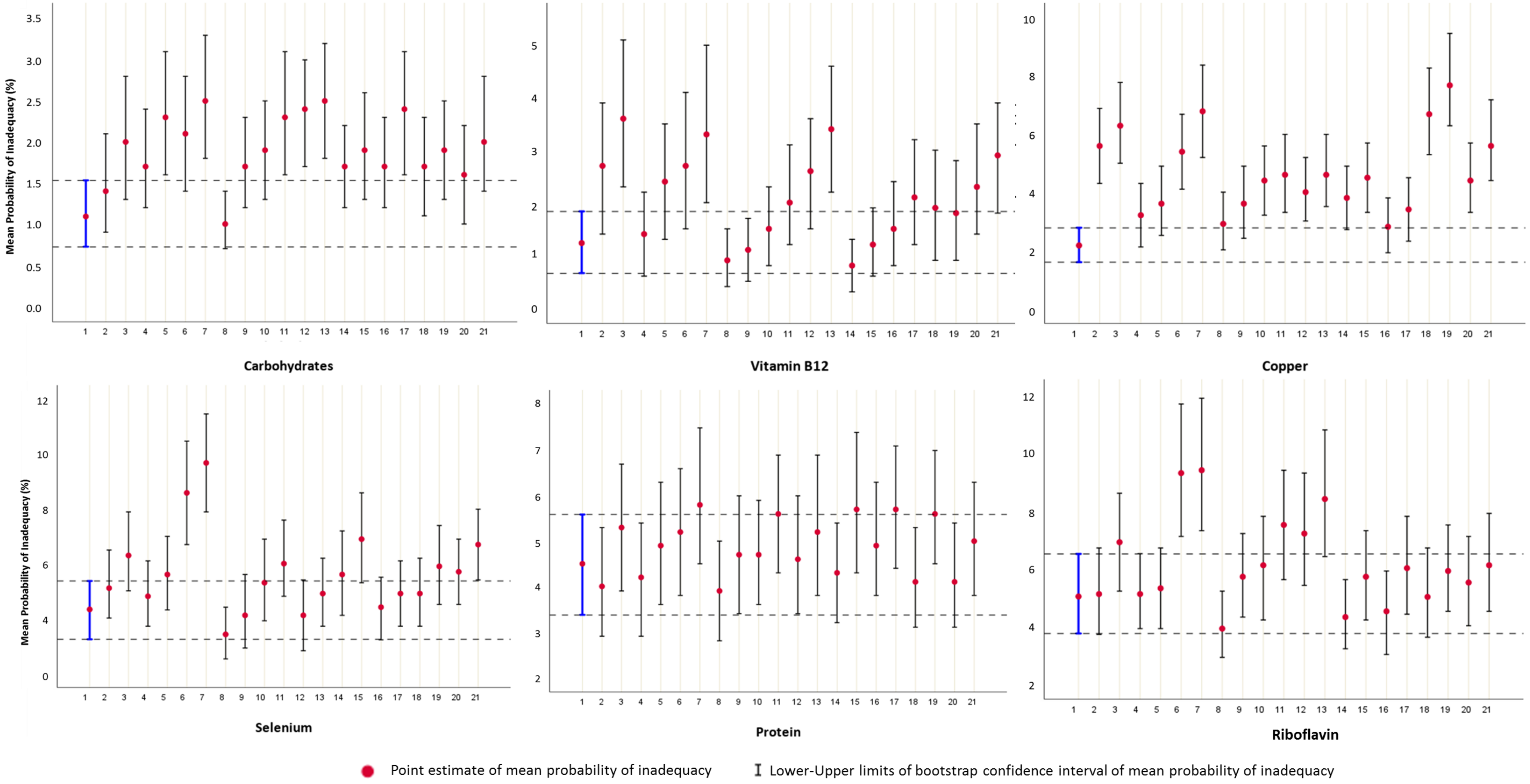

Supplementary Figure 5. Point estimates and bootstrap confidence intervals for the mean probability of inadequacy (%) for carbohydrates, vitamin B12, copper, selenium, protein and riboflavin. The estimates were calculated for: **1.** usual intake (blue line), and simulated datasets generated by: **2-3.** the mean and standard deviation of the original values, **4-10.** the combinations of lower and upper confidence limits of mean and standard deviation values of usual intakes, **11-21.** random combinations of mean and standard deviation values of usual intakes, within the confidence interval limits of usual intake. Odd numbers: Scenarios without considering the uncertainty into the model; Even numbers: Scenarios with considering the uncertainty into the model.

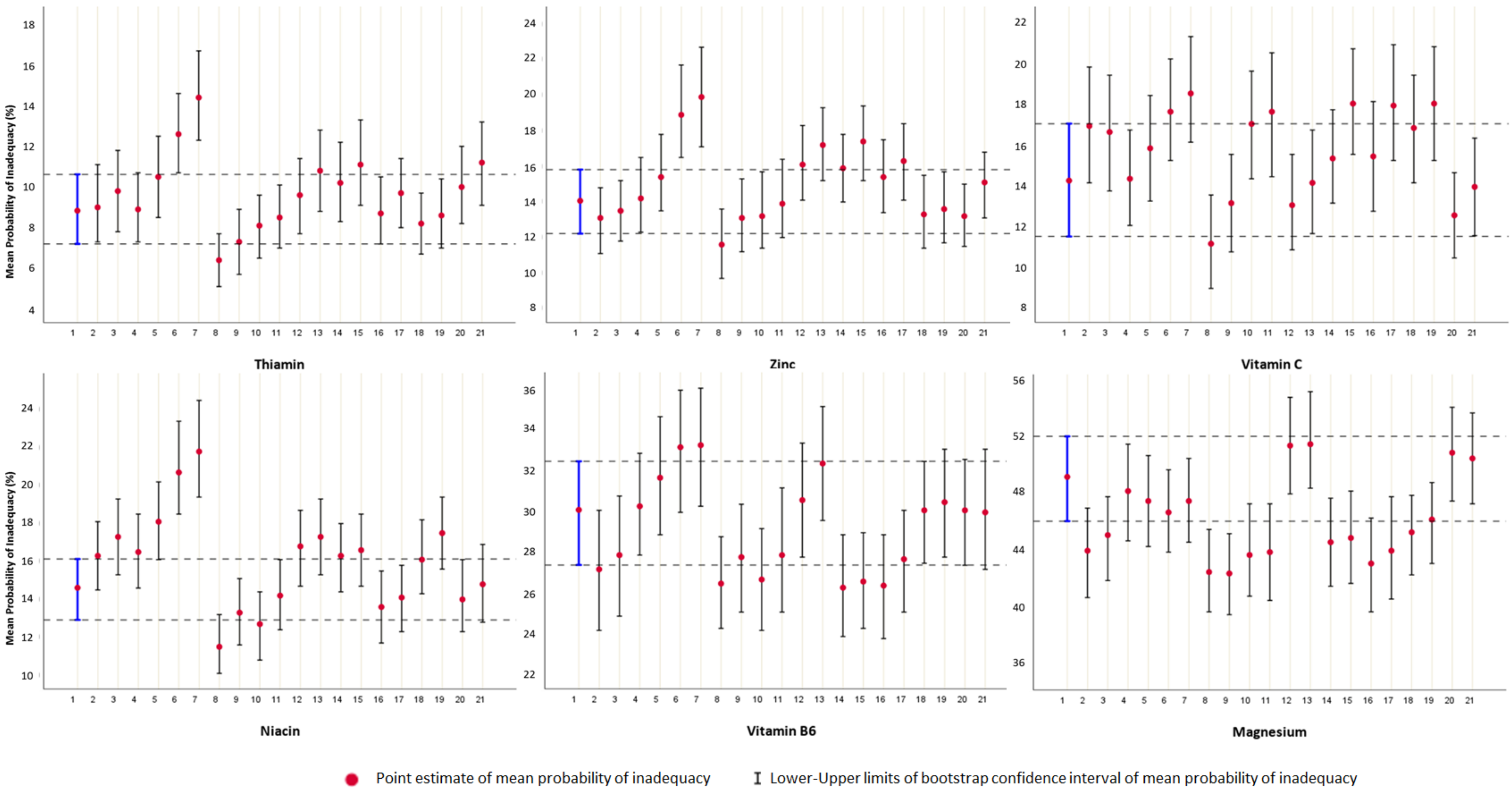

Supplementary Figure 6. Point estimates and bootstrap confidence intervals for the mean probability of inadequacy (%) for thiamin, zinc, vitamin C, niacin, vitamin B6 and magnesium. The estimates were calculated for: **1.** usual intake (blue line), and simulated datasets generated by: **2-3.** the mean and standard deviation of the original values, **4-10.** the combinations of lower and upper confidence limits of mean and standard deviation values of usual intakes, **11-21.** random combinations of mean and standard deviation values of usual intakes, within the confidence interval limits of usual intake. Odd numbers: Scenarios without considering the uncertainty into the model; Even numbers: Scenarios with considering the uncertainty into the model.

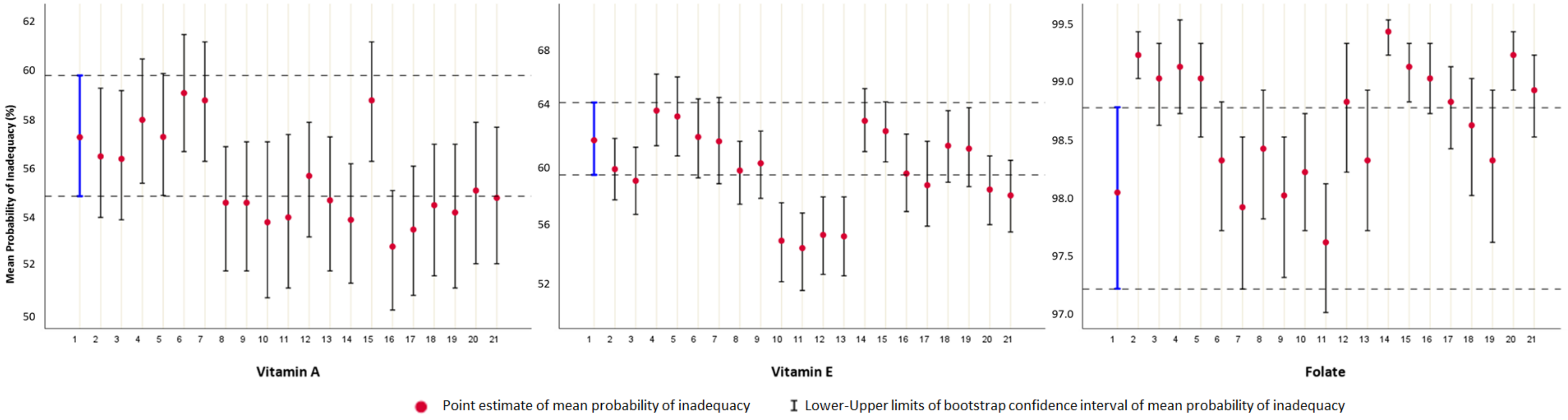

Supplementary Figure 7. Point estimates and bootstrap confidence intervals for the mean probability of inadequacy (%) for vitamin A, vitamin E and folate. The estimates were calculated for: **1.** usual intake (blue line), and simulated datasets generated by: **2-3.** the mean and standard deviation of the original values, **4-10.** the combinations of lower and upper confidence limits of mean and standard deviation values of usual intakes, **11-21.** random combinations of mean and standard deviation values of usual intakes, within the confidence interval limits of usual intake. Odd numbers: Scenarios without considering the uncertainty into the model; Even numbers: Scenarios with considering the uncertainty into the model.
